# Effectiveness comparison of nirmatrelvir/ritonavir versus molnupiravir in COVID-19 patients with comorbidities in Taiwan: a multi-centre electronic health record study

**DOI:** 10.1186/s12879-025-12316-0

**Published:** 2025-12-11

**Authors:** Wen-Kuang Lin, Shwu-Jiuan Lin, Yu-Hui Chang, Fu-Der Wang, Phung-Anh Nguyen, Phan Thanh Phuc, Whitney Burton, Nguyen Thi Kim Hien, Chia-Chieh Lin, Carlos Shu-Kei Lam, To-Chi Ha, Christine Y. Lu, Chih-Wei Huang, Hsuan-Chia Yang, Shiue-Ming Lin, Chieh Yang, Li-Hsuan Wang, Jason C. Hsu

**Affiliations:** 1 School of Pharmacy, College of Pharmacy, Taipei Medical University, No.250, Wuxing St., Xinyi Dist., Taipei City, 110 Taiwan (R.O.C.); 2 Division of Infection Diseases, Department of Internal Medicine, Taipei Medical University Hospital, No. 252, Wuxing St, Xinyi District, Taipei City, 110 Taiwan (R.O.C.); 3 Institute of Public Health, National Yang-Ming Chiao-Tung University, No. 155, Sec. 2, Linong St., Beitou Dist., Taipei City, 112304 Taiwan (R.O.C.); 4 Clinical Data Center, Office of Data Science, Taipei Medical University, No.250, Wuxing St., Xinyi Dist., Taipei City, 110 Taiwan (R.O.C.); 5 Clinical Big Data Research Center, Taipei Medical University Hospital, Taipei Medical University, No. 252, Wuxing St, Xinyi District, Taipei City, 110 Taiwan (R.O.C.); 6 Research Center of Data Science on Healthcare Industry, College of Management, Taipei Medical University, 11 Fl., Biomedical Technology Building, 301 Yuantong Rd., Zhonghe Dist., New Taipei City, 235 Taiwan (R.O.C.); 7 Graduate Institute of Data Science, College of Management, Taipei Medical University, 11 Fl., Biomedical Technology Building, 301 Yuantong Rd., Zhonghe Dist., New Taipei City, 235 Taiwan (R.O.C.); 8 International Ph.D. Program in Biotech and Healthcare Management, College of Management, Taipei Medical University, 11 Fl., Biomedical Technology Building, 301 Yuantong Rd., Zhonghe Dist., New Taipei City, 235 Taiwan (R.O.C.); 9 Master Program in Global Health and Health Security, College of Public Health, Taipei Medical University, No.250, Wuxing St., Xinyi Dist., Taipei City, 110 Taiwan (R.O.C.); 10 School of Nutrition and Health Sciences, College of Nutrition, Taipei Medical University, No.250, Wuxing St., Xinyi Dist., Taipei City, 110 Taiwan (R.O.C.); 11 Department of Pharmacy, Taipei Veterans General Hospital, No.201, Sec. 2, Shipai Rd., Beitou District, Taipei City, 112 Taiwan (R.O.C.); 12 Emergency Department, Department of Emergency and Critical Care Medicine, Wan Fang Hospital, Taipei Medical University, No.111, Sec. 3, Xinglong Rd., Wenshan Dist., Taipei City, 116 Taiwan (R.O.C.); 13 Department of Emergency, School of Medicine, College of Medicine, Taipei Medical University, No.250, Wuxing St., Xinyi Dist., Taipei City, 110 Taiwan (R.O.C.); 14 Department of Emergency and Critical Care Medicine, Taipei Medical University Shuang Ho Hospital, No.291, Zhongzheng Rd., Zhonghe District, New Taipei City, 23561 Taiwan (R.O.C.); 15 Department of Emergency, School of Medicine, Taipei Medical University, No.250, Wuxing St., Xinyi Dist., Taipei City, 110 Taiwan (R.O.C.); 16 Department of Population Medicine, Harvard Medical School and Harvard Pilgrim Health Care Institute, 401 Park Dr, Boston, MA 02215 USA; 17 Kolling Institute, Faculty of Medicine and Health, The University of Sydney and the Northern Sydney Local Health District, Reserve Rd, St Leonards, Sydney, NSW 2065 Australia; 18 School of Pharmacy, Faculty of Medicine and Health, The University of Sydney, A15, Science Road, Camperdown, Sydney, NSW 2050 Australia; 19 International Center for Health Information Technology (ICHIT), Taipei Medical University, No.250, Wuxing St., Xinyi Dist., Taipei City, 110 Taiwan (R.O.C.); 20 Graduate Institute of Biomedical Informatics, College of Medical Science and Technology, Taipei Medical University, No.250, Wuxing St., Xinyi Dist., Taipei City, 110 Taiwan (R.O.C.); 21 Research Center of Big Data and Meta-analysis, Wan Fang Hospital, Taipei Medical University, No.111, Sec. 3, Xinglong Rd., Wenshan Dist., Taipei City, 116 Taiwan (R.O.C.); 22 Department of Pharmacy, Taipei Medical University Hospital, No. 252, Wuxing St, Xinyi District, Taipei City, 110 Taiwan (R.O.C.)

**Keywords:** COVID-19, Nirmatrelvir/ritonavir, Molnupiravir, Comorbidity, Taipei medical university clinical research database (TMUCRD), Taiwan

## Abstract

**Background:**

COVID-19 patients frequently present with various comorbidities. Two developed antiviral medications, nirmatrelvir/ritonavir and molnupiravir, have been utilized in COVID-19 patients; but comparisons of the effectiveness between nirmatrelvir/ritonavir and molnupiravir in COVID-19 patients with different comorbidities remain unknown. This study aims to compare the effectiveness, including invasive ventilation and mortality, of nirmatrelvir/ritonavir and molnupiravir in the overall population and populations with various comorbidities in Taiwanese patients during the omicron BA.2 wave.

**Methods:**

We retrospectively collected electronic medical records from the Taipei Medical University Clinical Research Database between January and December 2022 and conducted an analysis of adult patients diagnosed with SARS-CoV-2 infection. For data management, we performed propensity score matching to minimize the imbalance between two groups; the standardized mean difference > 0.1 or a p value < 0.05 considered statistically significant. Variables, which remained imbalanced after matching, were adjusted by cox regression model. To identify the risk associated with these variables, a Cox proportional hazards model were performed. Kaplan-Meier method was applied to estimate invasive ventilation and mortality, comparing survival curves between nirmatrelvir/ritonavir users and molnupiravir users.

**Results:**

Our cohort was recruited from a database, including patients who receive nirmatrelvir/ritonavir or molnupiravir treatment. Out of a total of 35,617 patients, 968 patients received nirmatrelvir/ritonavir and 1198 patients received molnupiravir after matching. Patients with chronic liver disease or mental disease on nirmatrelvir/ritonavir had lower risks of intubation than those on molnupiravir. Overall, nirmatrelvir/ritonavir reduced mortality risk by 65% (adjusted hazard ratio (aHR): 0.35, 95% confidence interval (CI): 0.14–0.88, *p* = 0.026). For patients with diabetes mellitus (aHR: 0.29, 95% CI: 0.11–0.78, *p* = 0.014), with chronic kidney disease (aHR: 0.26, 95% CI: 0.10–0.68, *p* = 0.007), or aged over 65 years (aHR: 0.30, 95% CI: 0.13–0.70, *p* = 0.005), nirmatrelvir/ritonavir demonstrated superior efficacy in reducing mortality risk compared to molnupiravir.

**Conclusions:**

Data revealed that both nirmatrelvir/ritonavir and molnupiravir demonstrated clinical benefits in treating COVID-19 patients in a real-world setting. Moreover, nirmatrelvir/ritonavir was associated with a lower risk of mortality in COVID-19 patients with specific circumstances.

**Clinical trial:**

Clinical trial number is not applicable.

**Supplementary Information:**

The online version contains supplementary material available at 10.1186/s12879-025-12316-0.

## Introduction

Coronavirus disease 2019 (COVID-19) was first reported from China at the end of 2019 and exploded in Wuhan, China in January 2020 [1]. The World Health Organization (WHO) declared COVID-19 a global pandemic in 2020 which eventually impacted more than 200 countries, and there were over 770 million confirmed cases worldwide [2, 3]. COVID-19 is caused by severe acute respiratory syndrome (SARS)-coronavirus (CoV)-2 infection which can lead to multiple systemic manifestations. The major symptoms of patients with COVID-19 infection affect the respiratory system and include coughing, headaches, fever, shortness of breath [4].

There are two treatment strategies for managing COVID-19: vaccination and antiviral therapy. Different manufacturing methods of COVID-19 vaccines were developed, including virus-vectored vaccines, such as AZD1222 from Oxford/AstraZeneca and messenger (m)RNA vaccines, such as BNT162b2 from BNT/Pfizer and mRNA-1273 from Moderna; DNA vaccines and subunit vaccines were also reported. The effectiveness and well-tolerated safety profiles of these vaccines were demonstrated by clinical studies [5–7]. Over 150 vaccine candidates have so far been developed, and some of them are still under investigation [8].

Antiviral medications are another effective way to fight against COVID-19. Two novel antiviral medications, nirmatrelvir/ritonavir and molnupiravir, were evaluated by pivotal studies [9, 10]. Nirmatrelvir inhibits viral replication through inhibiting the main SARS-CoV-2 protease. Ritonavir, a human immunodeficiency virus (HIV)-1 protease inhibitor, inhibits the cytochrome P450, family 3, subunit A (CYP3A)-mediated metabolism of nirmatrelvir, leading to a pharmacokinetic change in nirmatrelvir [9]. Molnupiravir, a prodrug of ribonucleoside *N*-hydroxycytidine (NHC), exerts its antiviral effect by inhibiting RNA-dependent RNA polymerase, leading to errors in coronavirus replication [11]. Emergency use authorizations (EUAs) of nirmatrelvir/ritonavir and molnupiravir were approved by the Taiwanese Food and Drug Administration (FDA) in January 2022; nirmatrelvir/ritonavir received license approval in Taiwan in June 2023. For patients older than 12 years with mild to moderate COVID-19 and a high risk of progression to severe COVID-19, the approved dose of nirmatrelvir/ritonavir is 300 mg nirmatrelvir and 100 mg ritonavir twice daily for 5 days. This treatment course should be initiated after confirmation of a COVID-19 diagnosis and within 5 days of symptom onset. Molnupiravir can be used to manage adult patients with mild to moderate SARS-CoV-2 infection and who have a high risk of progression to severe COVID-19. The recommended dose is 800 mg molnupiravir every 12 h for 5 days. Initiation of the treatment course for molnupiravir has the same recommendations as nirmatrelvir/ritonavir. The relative effectiveness of nirmatrelvir/ritonavir and molnupiravir in COVID-19 patients with variable comorbidities has not been well established. In order to address this gap, therefore, we sought to compare the effectiveness of nirmatrelvir/ritonavir and molnupiravir in overall and specific populations with COVID-19 in Taiwanese patients during the omicron BA.2 wave from January to December 2022.

## Methods

### Study design and data source

We retrospectively collected and analyzed electronic medical record of patients aged at least 18 years with a COVID-19 diagnosis from the Taipei Medical University Clinical Research Database (TMUCRD) [12]. This is a comprehensive clinical database in which is collected integrated care information across virtual, outpatient, emergency department, and inpatient settings with over 4.1 million individual medical records from three medical institutes in northern Taiwan: Taipei Medical University Hospital (TMUH), Wan-Fang Hospital (WFH), and Shuang-Ho Hospital (SHH). Eligible participants were aged 18 years or older at the time of the index test, which was defined as the date of the first confirmed COVID-19 diagnosis, and had received a positive polymerase chain reaction (PCR) test result for SARS-CoV-2 between January 17 and December 31, 2022.

This study was approved by the Taipei Medical University Joint Institutional Review Board (TMU-JIRB), and informed consent was waived due to data anonymization and retrospective collection.

### Cohort selection

**Fig. 1:**
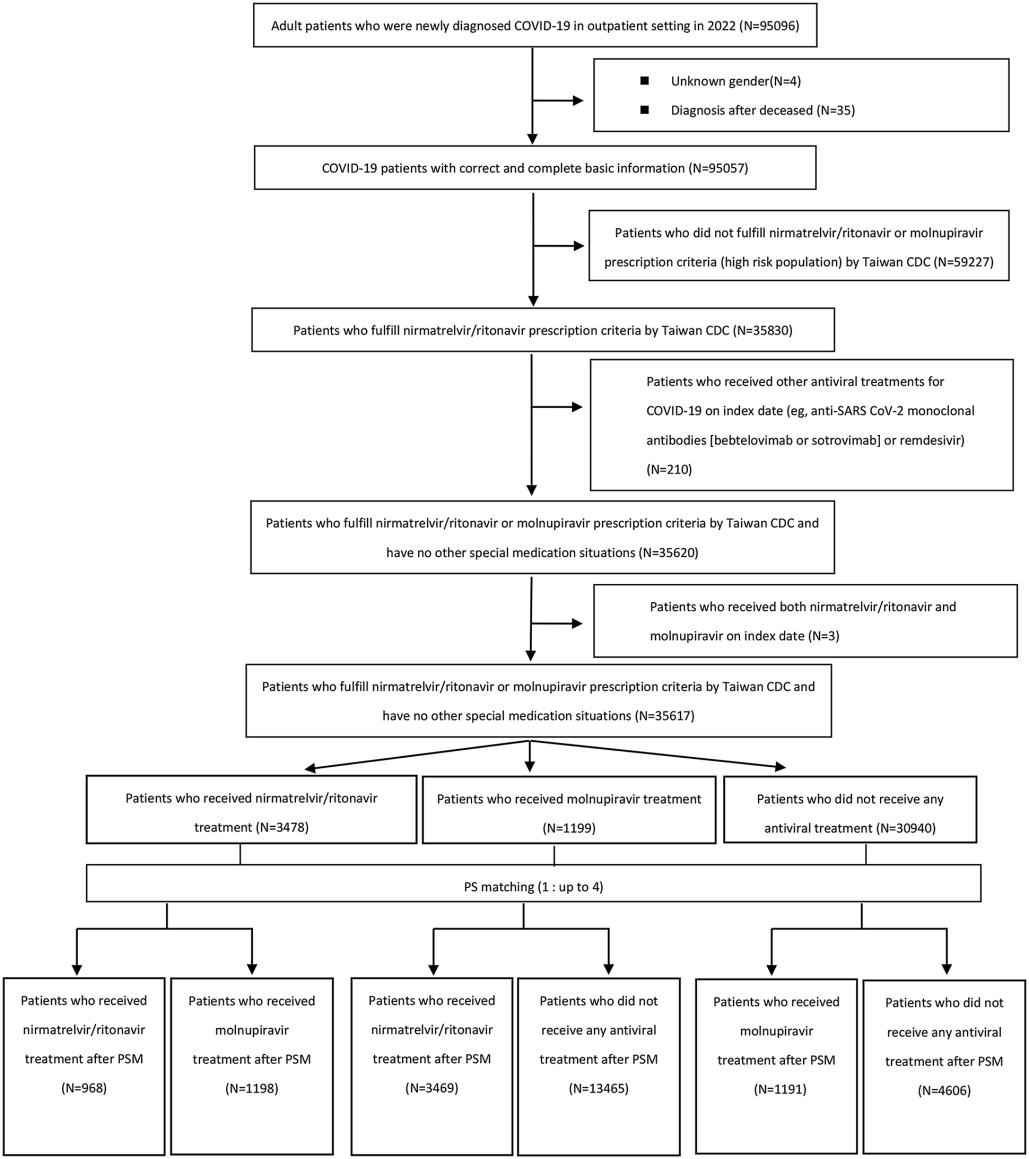
Flowchart of the cohort selection process. Notes: The patients were matched by age, gender, BMI, smoking, vaccination status, chronic kidney disease, chronic pulmonary disease, chronic liver disease, diabetes mellitus, asthma, cardiovascular disease, tuberculosis, immune disease (exclude HIV), dementia, mental illness and malignancies at baseline. CDC, centers for disease control; N, number; PS, propensity score; PSM, propensity score matching

The study included outpatients within the TMUCRD with a confirmed diagnosis of COVID-19 (ICD-10: U07.1) between January 17, 2022 (the approval date of the EUA of nirmatrelvir/ritonavir in Taiwan) and December 31, 2022. Subjects who fulfilled any of the following criteria were excluded from the study cohort: (1) patients on their initial visit to any of the the three hospitals (TMUH, WFH, and SHH), without prior medical records; (2) patients who did not fulfill nirmatrelvir/ritonavir prescription criteria by the Taiwanese Centers for Disease Control (CDC) (sTable 1 in Supplement 1); (3) patients who received other antiviral treatments for COVID-19 on the index date (e.g. anti-SARS CoV-2 monoclonal antibodies [bebtelovimab or sotrovimab] or remdesivir); and (4) those younger than 18 years. Electronic case data for these patients were retrieved, commencing on January 17, 2022, and extended retroactively to the inception of the TMUCRD on January 1, 2020. There were 95,096 patients from the TMUCRD who met the inclusion criteria; 59,479 patients were excluded, including four patients with an abnormal gender, 35 patients with a postmortem diagnosis, 59,227 patients who did not fulfill Taiwan CDC prescription criteria for nirmatrelvir/ritonavir or molnupiravir, and 213 patients who received combination therapies of nirmatrelvir/ritonavir and molnupiravir or other antiviral medications, such as bebtelovimab, sotrovimab, and remdesivir. In total, 35,617 patients were included in the study. Three subgroups were categorized: group 1 indicated patients who received nirmatrelvir/ritonavir; group 2 indicated patients who received molnupiravir; and group 3 indicated patients who did not receive any antiviral treatment (non-user). Furthermore, we performed the propensity score matching (PSM) at a ratio of up to 1:4 to develop three comparative analyses, including nirmatrelvir/ritonavir (*n* = 968) versus molnupiravir (*n* = 1,198), nirmatrelvir/ritonavir (*n* = 3,469) versus non-user (*n* = 13,465) and molnupiravir (*n* = 1,191) versus non-user (*n* = 4,606). Patients were matched by age, gender, body-mass index (BMI), smoking, vaccination status, chronic kidney disease (CKD), chronic pulmonary disease, chronic liver disease, diabetes mellitus (DM), asthma, cardiovascular disease (CVD), tuberculosis, immune disease (exclude HIV), dementia, mental illness and malignancies at baseline. Figure 1 illustrates the process of cohort selection.

### Outcome measurement

The main objective of this analysis was to evaluate the effectiveness of nirmatrelvir/ritonavir and molnupiravir in overall and specific populations in Taiwan. The comparison of the effectiveness between nirmatrelvir/ritonavir and molnupiravir included risks of invasive ventilator use and mortality; these two outcomes were measured within 30 days after COVID-19 diagnosis. Censoring rules are including loss of follow up and death within 30 days after COVID-19 diagnosis. For specific populations, we included COVID-19 patients with various comorbidities, such as type II DM, chronic pulmonary disease, CKD, chronic liver diseases, immune diseases (excluding HIV), CVD, and mental diseases. These comorbidities were identified in TMUCRD by the following definition: minimum of two outpatient visits or one hospitalization pertaining to the disease before the index date. Furthermore, we also included some background factors, including gender, age, and BMI when analyzing the effectiveness of nirmatrelvir/ritonavir and molnupiravir.

### Analysis of covariates

By consolidating an extensive literature review around a well-founded clinical viewpoint following consultations with physicians, we identified covariates potentially associated with intubation utilization and mortality among COVID-19 patients. The identified covariates included: (1) basic factors such as gender and age, BMI, and Charlson comorbidity index (CCI) score; (2) medical visitation history, such as the former patient status at TMU’s hospitals, defined by patients with medical visit history in TMU’s hospitals, having been vaccinated at a TMU hospital, and having had an emergency department visit or telemedicine consultation; and (3) prevalent comorbidities like DM, CKD, a CVD (myocardial infarction, congestive heart failure, peripheral vascular disease, or cerebrovascular disease), chronic pulmonary disease, immunodeficiency or immunosuppression, malignancy, tuberculosis, chronic liver disease, a mental disorder, or dementia.

We established comorbidity definitions through utilizing ICD-9 or ICD-10 disease codes presented in the records. Within the cohort, patients were classified as having comorbidities if their records indicated a minimum of two outpatient visits or one hospitalization pertaining to a disease before the index date. Comorbidities were considered, and the CCI score was calculated using disease codes from either the ICD-9 or ICD-10 classification systems found in medical records. For details on definitions of covariates and the CCI calculation method, see sTables 1 and 2 in Supplement 1.

### Statistical analysis

Baseline characteristics of the study cohort are presented as the mean and standard deviation (SD) for continuous variables and by the frequency and percentage for categorical variables. In this study, PSM was conducted using the PROC PSMATCH procedure in SAS. A greedy nearest-neighbor matching algorithm was applied with a 1:4 matching ratio (each treated subject matched to up to four controls) without replacement. To ensure adequate comparability, a caliper width of 0.2 standard deviations of the logit of the propensity score was used to restrict matches within a reasonable distance. The propensity score model included the following covariates: age, sex, baseline comorbidities, and relevant clinical characteristics, which were selected based on their clinical and methodological relevance to treatment assignment and outcomes. To establish discrepancies between the two groups, we employed the standardized mean difference (SMD) with a value of > 0.1 or a *p* value of < 0.05 considered statistically significant. In addition, variance ratios and propensity score distributions were examined to confirm balance. Graphical diagnostics, including Love plots and propensity score density plots, were generated to visually demonstrate improved balance after matching. Furthermore, we used the Cox regression model to adjust variables which remained imbalanced after matching (SMD > 0.1 or *p* < 0.05). A Cox proportional hazards model was used to estimate the hazard ratio (HR), accompanied by the 95% confidence interval (CI) and *p* value, which were reported to illustrate the risk associated with these variables. Kaplan-Meier method was also used to estimate the survival rate of invasive ventilation and mortality between nirmatrelvir/ritonavir users and molnupiravir users. Results were considered statistically significant when *p* values were < 0.05. All data management utilized SAS Enterprise Guide 9.4 software (SAS Institute, Cary, NC, USA).

## Results

After propensity score matching, 968 patients who received nirmatrelvir/ritonavir and 1198 patients who received molnupiravir were included in the final analysis. A flowchart of cohort selection is demonstrated in Fig. 1. Data showed that similar distributions of basic demographics between the nirmatrelvir/ritonavir and molnupiravir groups included gender, age, BMI, and most comorbidities, excluding the CCI score (nirmatrelvir/ritonavir: 2.12 ± 2.25, molnupiravir: 2.75 ± 2.59; SMD = 0.26, *p* < 0.001), DM (nirmatrelvir/ritonavir: 30.99%, molnupiravir: 35.81%; SMD = 0.1, *p* = 0.018), and CKD (nirmatrelvir/ritonavir: 15.91%, molnupiravir: 32.89%; SMD = 0.4, *p* < 0.001). An interesting observation was that the proportion of vaccinated patients under molnupiravir treatment was greater than that of patients who received nirmatrelvir/ritonavir treatment (nirmatrelvir/ritonavir vs. molnupiravir: 31.82% vs. 37.65%; SMD = 0.12, *p* = 0.005). On the other hand, we observed that patients aged ≥ 65 years had received more nirmatrelvir/ritonavir or molnupiravir than those younger than 65 years (Table 1). Basic characteristics of COVID-19 patients with/without nirmatrelvir/ritonavir and molnupiravir as well as further details of pre- and post-propensity score matching results (sTables 3–8), propensity score density plots (sFigure 1 A-F), and LOVE plots (sFigure 2 A-C) are given in Supplement 1.

**Table 1:** Basic characteristics of COVID-19 patients with comorbidities using nirmatrelvir/ritonavir or molnupiravir in 2022 after PSM

	COVID drugs^#^				SMD	*P* value
	Nirmatrelvir/Ritonavir		Molnupiravir			
	*N* = 968		*N* = 1198			
**Gender**					0.00	0.976
Female	462	47.73%	571	47.66%		
Male	506	52.27%	627	52.34%		
**Age**,** mean ± SD**	73.02 ± 12.60		72.91 ± 14.16		0.01	0.843
18 ~ 64	162	16.74%	275	22.95%		
>=65	806	83.26%	923	77.05%		
**BMI**,** mean ± SD**	24.73 ± 4.55		24.73 ± 5.08		0.00	0.988
<30	853	88.12%	1052	87.81%		
>=30	115	11.88%	146	12.19%		
**Former patient at TMU’s hospitals**					0.09	0.047
No	69	7.13%	61	5.09%		
Yes	899	92.87%	1137	94.91%		
**Vaccinated at TMU’s hospitals**					0.12	0.005
No	660	68.18%	747	62.35%		
Yes	308	31.82%	451	37.65%		
**Emergency Department**					0.06	0.189
No	292	30.17%	393	32.80%		
Yes	676	69.83%	805	67.20%		
**Telemedicine**					0.01	0.783
No	907	93.70%	1119	93.41%		
Yes	61	6.30%	79	6.59%		
**CCI score**,** mean ± SD**	2.12 ± 2.25		2.75 ± 2.59		0.26	< 0.001
**Comorbidities**						
Diabetes mellitus	300	30.99%	429	35.81%	0.10	0.018
Chronic kidney disease	154	15.91%	394	32.89%	0.40	< 0.001
Cardiovascular disease (excluding hypertension)	513	53.00%	679	56.68%	0.07	0.087
Chronic pulmonary disease	106	10.95%	152	12.69%	0.05	0.215
Immunodeficiency or immunosuppression	31	3.20%	54	4.51%	0.07	0.120
Malignancy	95	9.81%	118	9.85%	0.00	0.978
Tuberculosis	3	0.31%	8	0.67%	0.05	0.244
Chronic liver diseases	33	3.41%	50	4.17%	0.04	0.357
Mental disease	82	8.47%	87	7.26%	0.04	0.297
Dementia	129	13.33%	163	13.61%	0.01	0.850

# Using 1: up to 4 propensity score matching with matching variables including all variables in Table 1

BMI, body mass index; CCI, Charlson Comorbidity Index; HIV, human immunodeficiency virus; PSM, propensity score matching; SD, standard deviation; SMD, standardized mean difference; TMU, Taipei Medical University

Figure 2a shows the risk of intubation of COVID-19 patients with different comorbidities using nirmatrelvir/ritonavir or molnupiravir in 2022. In general, there was no significant difference in overall populations receiving these two anti-COVID-19 medications (adjusted HR (aHR): 0.51, 95% CI: 0.22–1.15, *p* = 0.106). On the other hand, at day 30, the survival rate of invasive ventilation was 98.0% for molnupiravir users and 99.1% for nirmatrelvir/ritonavir users (Log-rank test, *P* = 0.041; Fig. 2b). The specific nirmatrelvir/ritonavir population of COVID-19 patients, such as patients with a BMI of > 30 kg/m^2^, with chronic liver diseases, or with a mental disorder had a significantly lower risk of invasive ventilation compared to those receiving molnupiravir treatment. Both nirmatrelvir/ritonavir (aHR: 0.26, 95% CI: 0.17–0.40, *p* < 0.001) and molnupiravir (aHR: 0.54, 95% CI: 0.36–0.79, *p* = 0.002) groups of COVID-19 patients demonstrated clinical benefits compared to non-users (sFigs. 3 and 4 in Supplement 1).

**Fig. 2a:**
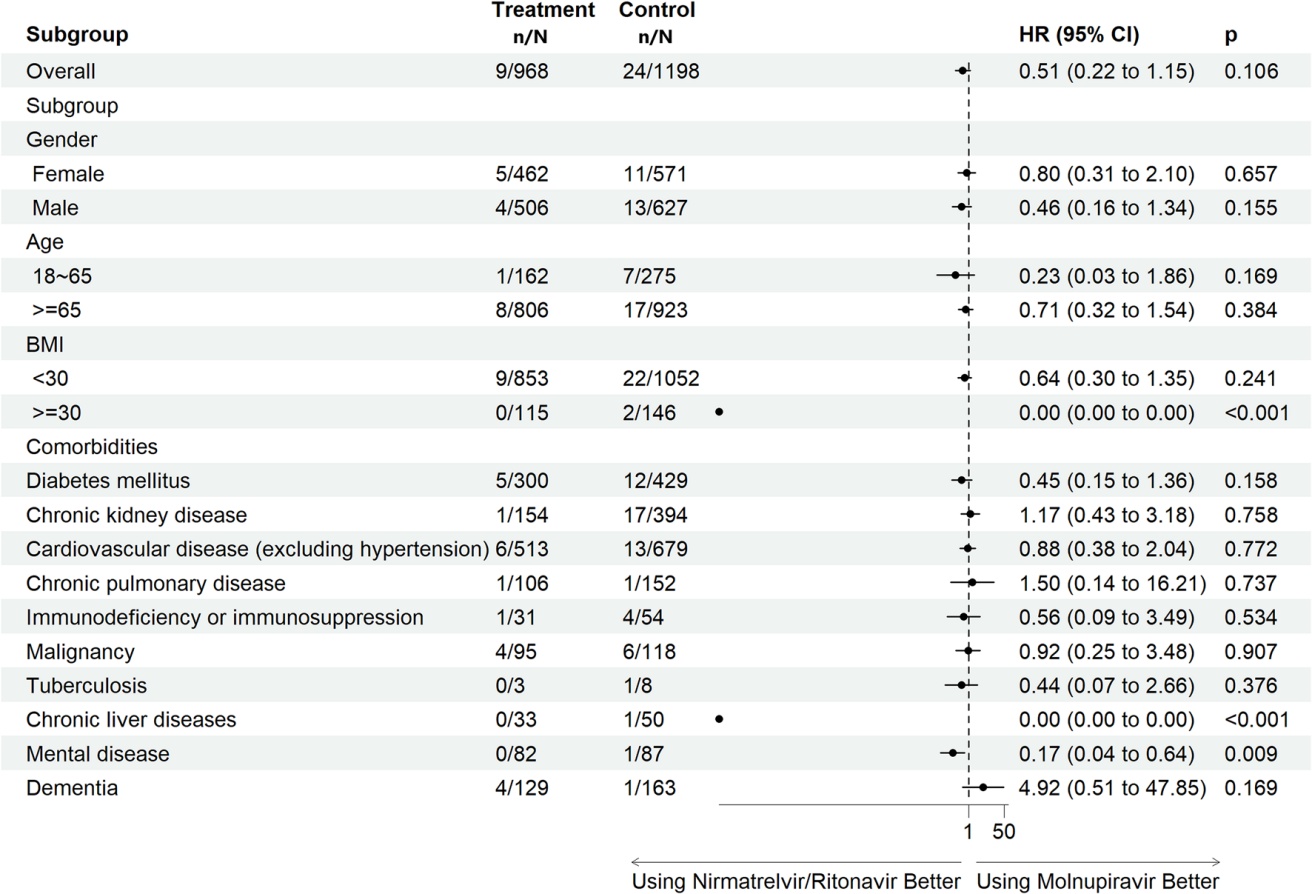
Risk of invasive ventilation in COVID-19 patients with comorbidities using nirmatrelvir/ritonavir or molnupiravir in 2022 [patients with inpatient prescription were excluded; outcomes within 30 days after COVID diagnosis]. Adjusted by age, CCI score, diabetes mellitus, chronic kidney disease, vaccinated or not at TMU’s hospitals and former patients or not TMU’s hospitals. Treatment group represents group of nirmatrelvir/ritonavir; control group represents group of molnupiravir. BMI, body mass index; CI, confidence interval; HR, hazard ratio; n, number (event number); N, number (treatment number); TMU: Taipei Medical University

**Fig. 2b:**
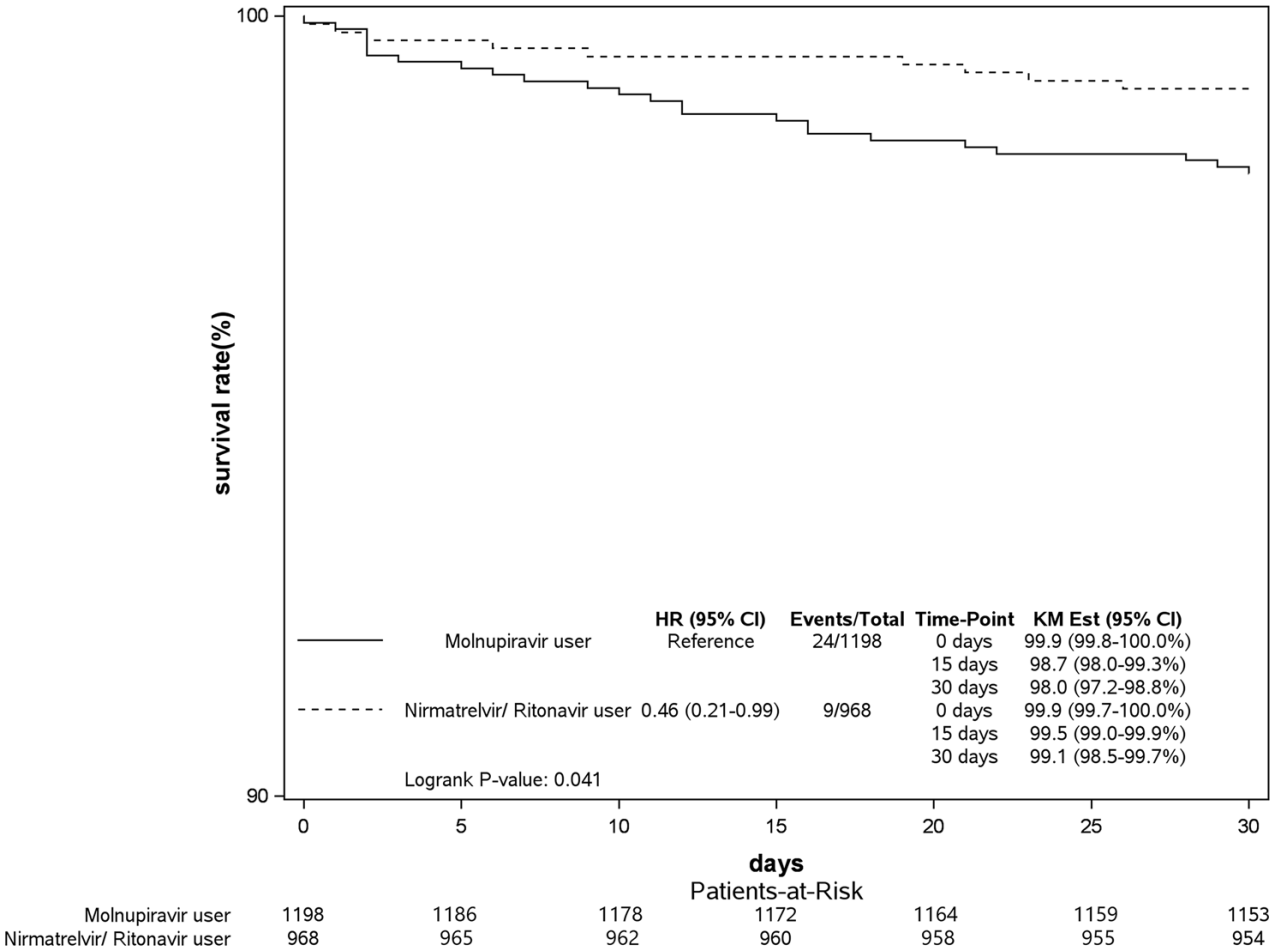
Kaplan–Meier curves of Invasive Ventilation in COVID-19 patients using nirmatrelvir/ritonavir or molnupiravir in 2022. KM Est, Kaplan-Meier Estimate

The aHRs of mortality for the nirmatrelvir/ritonavir and molnupiravir groups are shown in Fig. 3a. There was a 65% reduction in the risk of mortality in COVID-19 patients who received nirmatrelvir/ritonavir treatment (aHR: 0.35, 95% CI: 0.14–0.88, *p* = 0.026) compared to the molnupiravir group. The patients treated with molnupiravir showed a poorer survival rate than those treated with nirmatrelvir/ritonavir during the 30-day follow-up period (Log-rank test, *P* = 0.003; Fig. 3b). In addition, COVID-19 patients undergoing nirmatrelvir/ritonavir treatment with the following characteristics of female gender (aHR: 0.16, 95% CI: 0.04–0.71, *p* = 0.016), aged ≥ 65 years (aHR: 0.30, 95% CI: 0.13–0.70, *p* = 0.005), or comorbid with DM (aHR: 0.29, 95% CI: 0.11–0.78, *p* = 0.014) or CKD (aHR: 0.26, 95% CI: 0.10–0.68, *p* = 0.007) had lower risks of mortality. There was an association of a low risk of mortality for subjects in the nirmatrelvir/ritonavir group with CVDs, excluding hypertension, (aHR: 0.37, 95% CI: 0.13–1.01, *p* = 0.053). COVID-19 patients in both the nirmatrelvir/ritonavir (aHR: 0.15, 95% CI: 0.08–0.28, *p* < 0.001) and molnupiravir (aHR: 0.56, 95% CI: 0.37–0.84, *p* = 0.006) groups had reduced risks of mortality compared to non-users (sFigs. 5 and 6 in Supplement 1).

**Fig. 3a:**
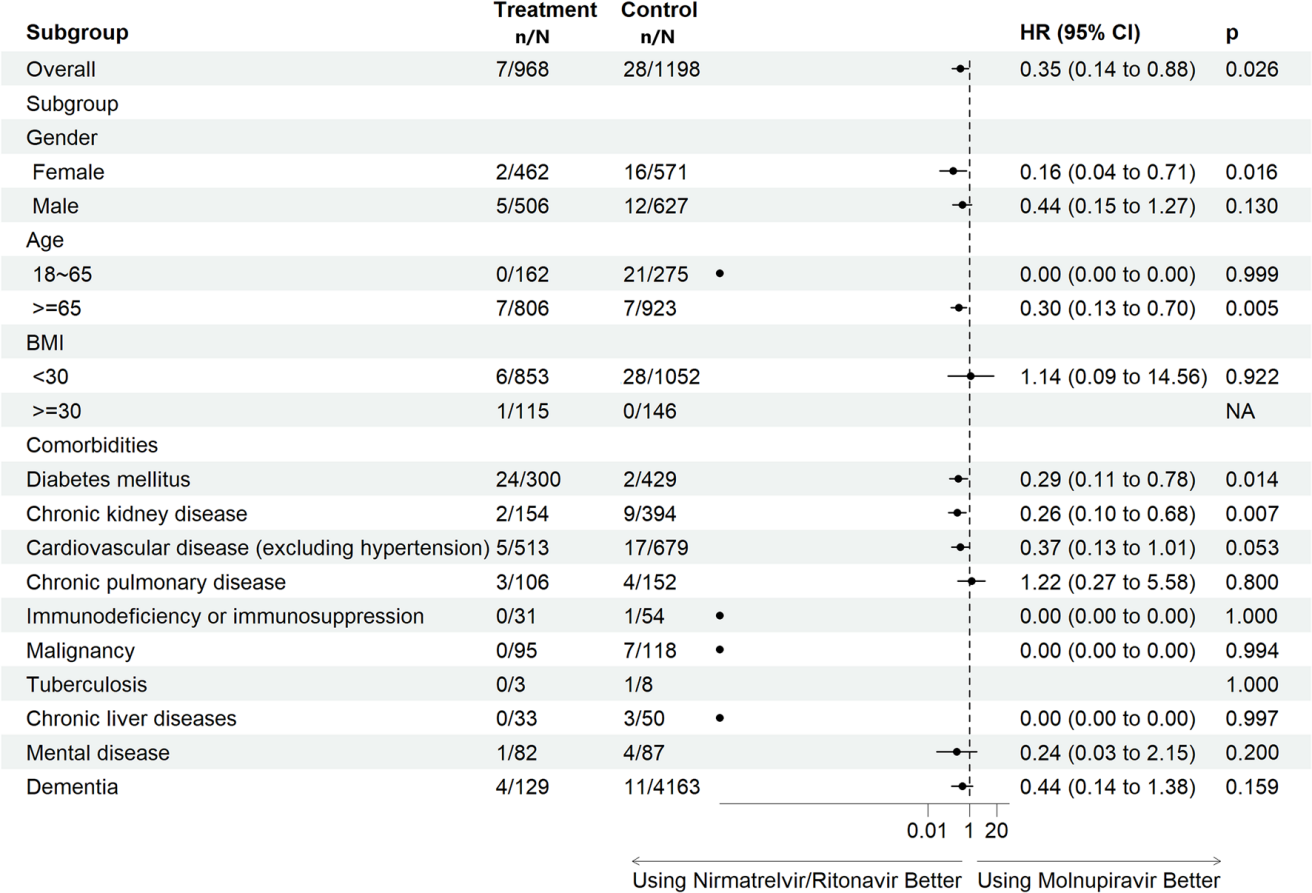
Risk of mortality in COVID-19 patients with comorbidities using nirmatrelvir/ritonavir or molnupiravir in 2022 [patients with inpatient prescription were excluded; outcomes within 30 days after COVID diagnosis]. Adjusted by age, CCI score, diabetes, chronic kidney disease, vaccinated or not at TMU’s hospitals and former patients or not TMU’s hospitals. Treatment group represents group of nirmatrelvir/ritonavir; control group represents group of molnupiravir. BMI, body mass index; CI, confidence interval; HR, hazard ratio; NA, not applicable; n, number (event number); N, number (treatment number); TMU: Taipei Medical University

**Fig. 3b:**
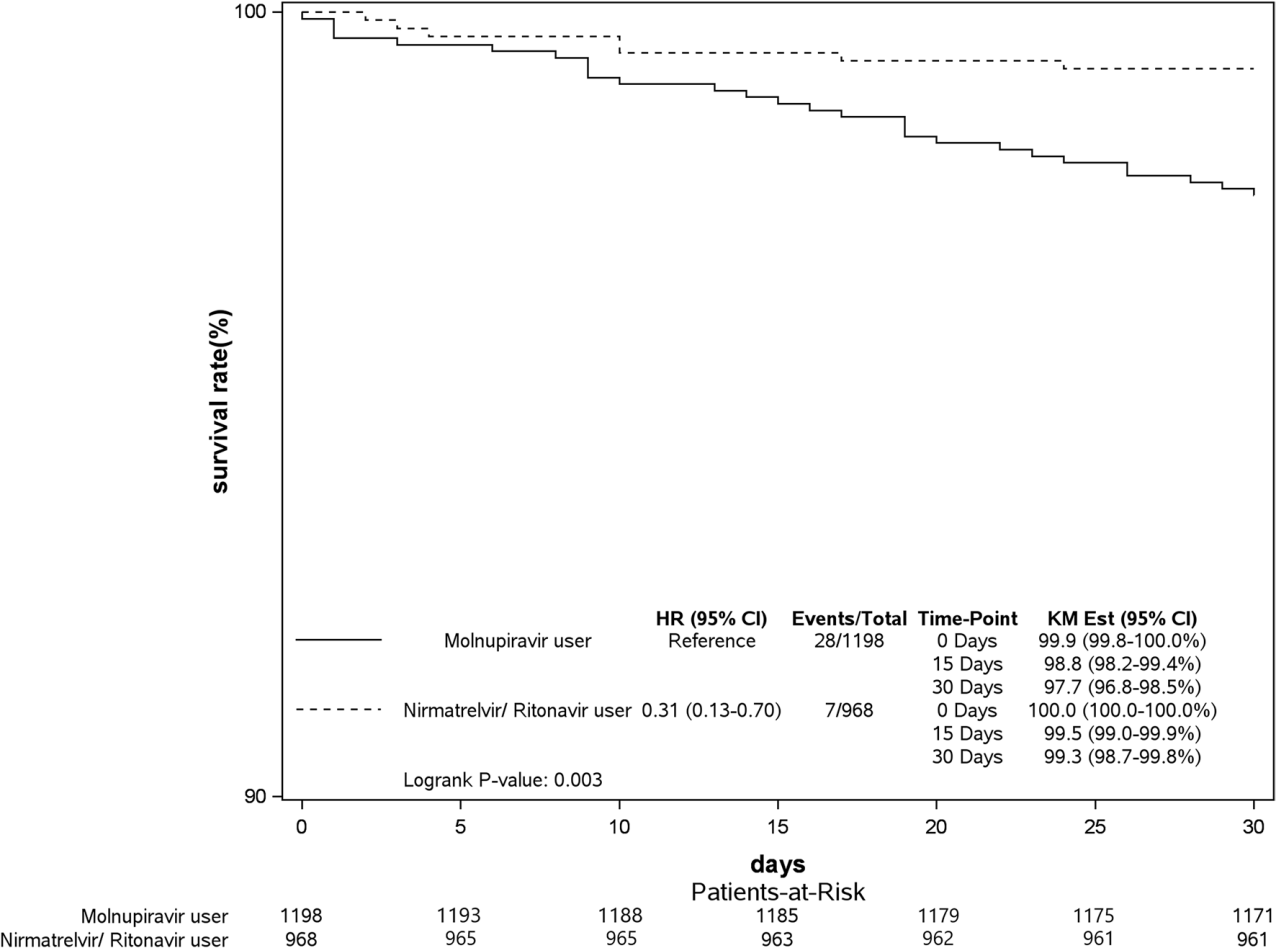
Kaplan–Meier curves of mortality in COVID-19 patients using nirmatrelvir/ritonavir or molnupiravir in 2022. KM Est, Kaplan-Meier Estimate

## Discussion

In this retrospective, multicenter study, we used data from the TMUCRD to evaluate the effectiveness of nirmatrelvir/ritonavir and molnupiravir in Taiwanese COVID-19 patients with various comorbidities. This analysis demonstrated several important observations in these two novel anti-COVID-19 medications.

First, utilization rates of anti-COVID-19 medications in patients aged over 65 years were at least twice those aged under than 65 years in the nirmatrelvir/ritonavir and molnupiravir groups. Compared to the utilization rate of anti-COVID-19 medications in the United States, the utilization share was higher in older patients in the molnupiravir group; on the other hand, data demonstrated that patients older than 85 years received comparatively lower nirmatrelvir/ritonavir treatment than the younger population because nirmatrelvir/ritonavir leads to several drug-drug interactions [13]. EUAs of nirmatrelvir/ritonavir and molnupiravir were approved by the Taiwanese FDA in January 2022, and at the same time, the Taiwanese CDC announced that nirmatrelvir/ritonavir and molnupiravir treatment would be reimbursed by the government for COVID-19 patients who met the medication label requirements and other related criteria. These criteria included being aged over 65 years, having certain comorbidities (malignancy, DM, CKD, immune diseases, etc.), and being overweight, defined as a BMI of ≥ 30 kg/m^2^ [14]. This illustrates why there were higher prescription rates of nirmatrelvir/ritonavir and molnupiravir in adults aged ≥ 65 years. On the other hand, it was notable that around 30% of COVID-19 patients had received a COVID-19 vaccination in our cohort study. Low vaccination rates in the two groups were due to the study definition. In our definition, we only tracked patients who had received a COVID-19 vaccine in the three affiliated hospitals; there were, however, 87% of individuals aged ≥ 6 months had received their first dose by the end of 2022, as reported by the Taiwanese CDC [15]. Therefore, we could assume that most study participants had received at least one dose of the COVID-19 vaccine in the study period.

Second, evidence suggested that nirmatrelvir/ritonavir and molnupiravir were both effective in inhibiting viral replication and reducing hospitalization and mortality rates [16–20]. Our data showed that both the nirmatrelvir/ritonavir and molnupiravir groups demonstrated clinical benefits in the overall COVID-19 population compared to non-users. There were 74% and 46% risk reductions of intubation and 85% and 44% risk reductions in mortality, respectively, for the nirmatrelvir/ritonavir and molnupiravir groups. In addition, a recent publication of a systematic review and meta-analysis of observational studies demonstrated the superior efficacy of several perspectives of nirmatrelvir/ritonavir over molnupiravir, including mortality and hospitalization rates and negative PCR conversion times [21]. Other research found that both molnupiravir and nirmatrelvir/ritonavir accelerated SARS-CoV-2 viral clearance in the oral airway in COVID-19 patients, but the antiviral effect of nirmatrelvir/ritonavir was substantially greater [22]. For the overall population, our results are generally consistent with previous findings that nirmatrelvir/ritonavir might have better efficacy than molnupiravir in reducing the risk of mortality. Although the reduction in the risk of invasive ventilation was not statistically significant, patients treated with nirmatrelvir/ritonavir still had a lower trend of intubation than those treated with molnupiravir.

Third, previous publications noted that nirmatrelvir/ritonavir was associated with lower risks of hospital admission, intubation, and mortality in COVID-19 patients with various comorbidities, such as CKD [23], CVDs [24, 25], DM [24–26], immunosuppression [24, 27, 28], malignancies [25, 29], and non-alcoholic fatty liver disease [30]. Furthermore, similar results for molnupiravir were observed in the available literature [25, 26, 30–33]. One retrospective study explored how early initiation of nirmatrelvir/ritonavir significantly reduced risks of invasive ventilation, intensive care unit (ICU) admission, and all-cause mortality in COVID-19 patients with severe CKD [23]. Data analysis from a cohort study conducted in Israel demonstrated that nirmatrelvir/ritonavir was effective in reducing the threats of severe COVID-19 progression and mortality in COVID-19 patients with CVD [24]. Another retrospective cohort study from the TriNetX US collaborative network supported that both the nirmatrelvir/ritonavir and molnupiravir groups had lower risks of all-cause hospitalization and mortality in patients comorbid with COVID-19 and CVD [25]. Research from Hong Kong revealed that these two oral antiviral medications were connected to lower risks of hospitalization and all-cause mortality among COVID-19 patients with type 2 DM [26]. For immunocompromised individuals, both Dormuth et al.’s and Javed et al.’s studies showed that nirmatrelvir/ritonavir diminished the risks of hospitalization and mortality [27, 28]. A study by Salmanton-Garcia et al., which explored hematological malignancy patients with extrapulmonary symptoms such as anosmia, fever, rhinitis, or sinusitis at COVID-19 onset, found that they were more prone to receive nirmatrelvir/ritonavir compared to subjects with chronic pulmonary disease or obesity [29]. In addition, similar results were observed in molnupiravir’s real-world evidence, conducted by Bolkun et al. [32]. For individuals with non-alcoholic fatty liver disease, data depicted low risks of all-cause emergency department visits, hospitalizations, and mortality in COVID-19 patients receiving nirmatrelvir/ritonavir or molnupiravir [30]. Our results, which were consistent with the aforementioned studies, showed that nirmatrelvir/ritonavir had lower risks in invasive ventilation in COVID-19 patients with various comorbidities, including DM, CKD, CVDs (excluding hypertension), chronic pulmonary disease, chronic liver disease, and mental disease. As to reducing the risk of mortality, nirmatrelvir/ritonavir conferred benefits to COVID-19 patients with DM, CKD, CVDs (excluding hypertension), chronic pulmonary disease, and dementia. On the other hand, treating COVID-19 patients comorbid with DM, CKD, or CVDs (excluding hypertension) with molnupiravir was associated with a reduced risk of intubation; as for reduced mortality risks, only patients with CVDs (excluding hypertension) receiving molnupiravir were observed compared to those with other comorbidities.

Lastly, in terms of a comparison of the efficacy between nirmatrelvir/ritonavir and molnupiravir in individuals with related comorbidities, numerous publications revealed that compared to molnupiravir, COVID-19 patients with advanced kidney disease or type 2 DM under nirmatrelvir/ritonavir treatment had lower rates of hospital admissions and all-cause mortality [34, 35]. Other literature showed that molnupiravir was comparable to nirmatrelvir/ritonavir in preventing progression to severe disease/mortality and had a superior safety profile in cancer patients with COVID-19 [36]. In line with previous studies, our data suggested that COVID-19 patients with DM or CKD who received nirmatrelvir/ritonavir had a lower risk of mortality compared to those with molnupiravir. Furthermore, new findings from our analysis demonstrated that, compared to molnupiravir users, nirmatrelvir/ritonavir conferred a lower risk of intubation in subjects with chronic liver disease or mental disease.

This study has several strengths and limitations. In terms of strengths, it is the first multi-center real-world study comparing nirmatrelvir/ritonavir and molnupiravir in COVID-19 patients with different comorbidities. There were a few cases of COVID-19 infection before the omicron BA.2 subtype outbreak in Taiwan in 2022 [37]; therefore, our data, compared to current available studies, demonstrated the true efficacy of these two oral antiviral drugs in COVID-19 patients with pure omicron subtype infection. Our results support the efficacy of these two antiviral drugs compared to each other and can assist physicians in choosing the right medications for particular patients. Some limitations, however, were evident in our study. The main limitation was the retrospective design of the study and its inclusion of only three tertiary referral hospitals in northern Taiwan. Some clinical information might have been lacking or there may have been recording bias, such as for vaccination status, phase of illness, days of symptom onset and viral load; therefore interpretation should be done with caution. Although we performed propensity score matching and followed by cox regression to minimize the risk of imbalance, residual confounding might persist and should be acknowledged. Another limitation was that physicians were following Taiwanese CDC guidelines for treating COVID-19 patients with high risk factors, which may have produced selection bias in patients with anti-COVID-19 medications. While this study provides initial insights, caution must be exercised when interpreting causal relationships. The causal inferences need to be validated by future studies.

## Conclusions

In conclusion, both nirmatrelvir/ritonavir and molnupiravir treatments conferred clinical benefits in COVID-19 patients of reducing risks of invasive ventilation and mortality. In terms of specific populations, nirmatrelvir/ritonavir users with chronic liver disease or a mental disease had significantly lower rates of invasive ventilation compared to the molnupiravir cohort. On the other hand, COVID-19 patients with DM or CKD or female gender or aged over 65 year who received nirmatrelvir/ritonavir, had a lower risk of mortality compared to the molnupiravir cohort. Physicians might consider prioritizing treatment of nirmatrelvir/ritonavir for those patient groups to achieve better outcomes. The reduction in the risk of mortality between the nirmatrelvir/ritonavir and molnupiravir cohorts exhibited no significant differences for other comorbidities. In general, the available evidence and our data suggest that nirmatrelvir/ritonavir and molnupiravir are effective treatments against COVID-19.

## Supplementary Information

Below is the link to the electronic supplementary material.

Supplementary Material 1

Supplementary Material 2

## Data Availability

All data generated or analysed during this study are included in this published article and its supplementary information files.

## Abbreviations

aHR: Adjusted Hazard Ratio
BMI: Body Mass Index
CCI: Charlson Comorbidity Index
CDC: Centers for Disease Control
CI: Confidence Interval
CKD: Chronic Kidney Disease
COVID-19: Coronavirus disease 2019
CVDs: Cardiovascular Diseases
CYP3A: Cytochrome P450, family 3, subunit A
DM: Diabetes Mellitus
DNA: Deoxyribonucleic Acid
EUAs: Emergency Use Authorizations
FDA: Food and Drug Administration
HIV: Human Immunodeficiency Virus
HR: Hazard Ratio
ICD-9: International Classification of Diseases, Ninth Revision
ICD-10: International Classification of Diseases, Tenth Revision
ICU: Intensive Care Unit
KM Est: Kaplan-Meier Estimate
mRNA: Messenger Ribonucleic Acid
NHC: N-hydroxycytidine
PCR: Polymerase Chain Reaction
SARS-CoV-2: Severe Acute Respiratory Syndrome – Coronavirus − 2
SD: Standard Deviation
SHH: Shuang-Ho Hospital
SMD: Standardized Mean Difference
TMU: Taipei Medical University
TMUCRD: Taipei Medical University Clinical Research Database
TMUH: Taipei Medical University Hospital
TMU-JIRB: Taipei Medical University Joint Institutional Review Board
WFH: Wan-Fang Hospital
WHO: World Health Organization
