# Supplementary material for: Effectiveness comparison of nirmatrelvir/ritonavir versus molnupiravir in COVID-19 patients with comorbidities in Taiwan: a multi-centre electronic health record study
Source: BMC Infect Dis. 2025 Dec 11;26:70. doi: 10.1186/s12879-025-12316-0 (PMC12801939; doi:10.1186/s12879-025-12316-0)
Supplement: Supplementary file 1 — Supplementary Material 1 [file 12879_2025_12316_MOESM1_ESM.docx]

**Supplement Online Content**

| **sTable 1** | **Definitions of Covariates** |
| --- | --- |
| **sTable 2** | **CCI Calculation Method** |
| **sTable 3** | **Basic Characteristics of COVID-19 Patients with Comorbidities Using Nirmatrelvir/Ritonavir or Not in 2022 before PSM** |
| **sTable 4** | **Basic Characteristics of COVID-19 Patients with Comorbidities Using Molnupiravir or Not in 2022 before PSM** |
| **sTable 5** | **Basic Characteristics of COVID-19 Patients with Comorbidities Using Nirmatrelvir/Ritonavir or Molnupiravir in 2022 before PSM** |
| **sTable 6** | **Basic Characteristics of COVID-19 Patients with Comorbidities Using Nirmatrelvir/Ritonavir or Not in 2022 after PSM** |
| **sTable 7** | **Basic Characteristics of COVID-19 Patients with Comorbidities Using Molnupiravir or Not in 2022 after PSM** |
| **sTable 8** | **Variance Ratios of Baseline Characteristics of COVID-19 Patients with Comorbidities Using Nirmatrelvir/Ritonavir, Molnupiravir or Not** |
| **sFigure 1** | **Density Plots of Propensity Scores Before and After Matching in COVID-19 Patients Using Nirmatrelvir/Ritonavir, Molnupiravir or Not in 2022** |
| **sFigure 2** | **LOVE Plots Demonstrated the Absolute Standardized Mean Differences on the Matching Variables Between Nirmatrelvir/Ritonavir and Molnupiravir.** |
| **sFigure 3** | **Risk of Invasive Ventilation in COVID-19 Patients with Comorbidities Using Nirmatrelvir/Ritonavir or Not in 2022** |
| **sFigure 4** | **Risk of Invasive Ventilation in COVID-19 Patients with Comorbidities Using Molnupiravir or Not in 2022** |
| **sFigure 5** | **Risk of Mortality in COVID-19 Patients with Comorbidities Using Nirmatrelvir/Ritonavir or Not in 2022** |
| **sFigure 6** | **Risk of Mortality in COVID-19 Patients with Comorbidities Using Molnupiravir or Not in 2022** |

**This supplemental material has been provided by the authors to give readers additional information about this study.**

**sTable 1. Definitions of Covariates**

| **Variable** | **Operational definition** | **Note** |
| --- | --- | --- |
| Age | Age at the index date in years.  As recorded in medical record or calculated as (Year of index date - birth year) Age will also be grouped into age groups (e.g., <64, 65+) |  |
| Former patient at TMU’s hospitals | ever a patient at TMU's hospitals or not | Before index date |
| Vaccinated at TMU’s hospitals | ever vaccinated at TMU's hospitals | Minimum of two outpatient visits or one hospitalization pertaining to the disease before the index date. (not including index date) |
| Emergency Department | COVID was diagnosed by TMU's ER |  |
| Telemedicine | COVID was diagnosed by telemedicine |  |
| Diabetes mellitus | ICD-10-CM code for diabetes: E10.x-E14.x | Minimum of two outpatient visits or one hospitalization pertaining to the disease before the index date. (not including index date) |
| Chronic kidney disease | ICD-10-CM code for chronic kidney disease: I12.x, I13.x, K76.7, N18.x, P96.0, Z99.2 | Minimum of two outpatient visits or one hospitalization pertaining to the disease before the index date. (not including index date) |
| Cardiovascular disease (excluding hypertension) | ICD-10-CM code for cardiovascular disease (excluding hypertension):  I00.x-I09.x, I20.x-I45.x, I47.x-I52.x, I60.x-I69.x, I70.x-I99.x | Minimum of two outpatient visits or one hospitalization pertaining to the disease before the index date. (not including index date) |
| Chronic pulmonary disease | Chronic pulmonary disease and its ICD-10-CM code includes:  Interstitial lung disease: J60.x-J67.x, J68.4, J70.1, J70.3, J84.x Pulmonary embolism: I26.x Pulmonary hypertension: I27.0 Bronchiectasis: J47.x Chronic obstructive pulmonary disease (COPD): J40.x, J41.x, J42.x, J43.x, J44.x | Minimum of two outpatient visits or one hospitalization pertaining to the disease before the index date. (not including index date) |
| Immunodeficiency or immunosuppression | Immunodeficiency or immunosuppression and its ICD-10-CM code includes: Human immunodeficiency virus (HIV): B20.x–B22.x, B24.x Primary immunodeficiencies: D70.x, D71.x, D80.x-D84.x, D89.x Solid organ or hematopoietic stem cell transplantation: T86x, Z94.0-Z94.4, Z94.81-Z94.84, Z94.89, Z94.9 Use of corticosteroids or other immunosuppressive medications: Z79.52, Z92.21, Z92.241, Z92.25 | Minimum of two outpatient visits or one hospitalization pertaining to the disease before the index date. (not including index date) |
| Malignancy | ICD-10-CM code for malignancy: C00.x–C26.x, C30.x–C34.x, C37.x–C41.x, C43.x, C45.x–C58.x, C60.x–C76.x, C77.x–C80.x, C97.x | Minimum of two outpatient visits or one hospitalization pertaining to the disease before the index date. (not including index date) |
| Tuberculosis | ICD-10-CM code for tuberculosis: A15.x | Minimum of two outpatient visits or one hospitalization pertaining to the disease before the index date. (not including index date) |
| Chronic liver diseases | Chronic liver diseases and its ICD-10-CM code includes:  Cirrhosis: K74.x Non-alcoholic fatty liver disease: K75.81, K76.0 Alcoholic liver disease: K70.x Autoimmune hepatitis: K75.4 | Minimum of two outpatient visits or one hospitalization pertaining to the disease before the index date. (not including index date) |
| Mental disease | Mental health conditions and its ICD-10-CM code includes:  Mood disorders: F30.x-F34.x, F39.x Schizophrenia spectrum disorders: F20.x, F22.x, F23.x, F25.x, F28.x, F29.x | Minimum of two outpatient visits or one hospitalization pertaining to the disease before the index date. (not including index date) |
| Dementia | ICD-10-CM code for dementia: F01.x-F03.x, G31.0, G31.83 | Minimum of two outpatient visits or one hospitalization pertaining to the disease before the index date. (not including index date) |

TMU, Taipei Medical University

**sTable 2. CCI Calculation Method**

| **Disease** | **ICD9-CM** | **ICD10-CM** | **Weight** |
| --- | --- | --- | --- |
| Myocardial infarction | 410, 412 | I21, I22, I23 | 1 |
| Congestive heart failure | 39891, 40201, 40211, 40291, 40401, 40403, 40411, 40413, 40491, 40493, 4254, 4255, 4257, 4258, 4259, 428 | I43, I50, I099, I110, I130, I132, I255, I420, I425, I426, I427, I428, I429, P290 | 1 |
| Peripheral artery disease | 0930, 4373, 440, 441, 4431, 4432, 4438, 4439, 4471, 5571, 5579,V434 | I70, I71, I731, I738, I739, I771, I790, I792, K551 , K558, K559, Z958, Z959 | 1 |
| Cerebrovascular Disease | 36234, 430, 431, 432, 433, 434, 435, 436, 437, 438 | G45, G46, I60, I61, I62, I63, I64, I65, I66, I67, I68, I69, H340 | 1 |
| Dementia | 290, 2941, 3312 | F00, F01, F02, F03, G30, F051, G311 | 1 |
| COPD | 4168, 4169, 490, 491, 492, 493, 494, 495, 496, 500, 501, 502, 503, 504, 505, 5064, 5081, 5088 | , J40, J41, J42, J43, J44, J45, J46, J47, J60, J61, J62, J63, J64, J65, J66, J67, I278, I279, J684, J701, J703 | 1 |
| Connective tissue disease | 4465, 7100, 7101, 7102, 7103, 7104, 7140, 7141, 7142, 7148, 725 | M05,M32,M33,M34,M06,M315,M351,M353,M360 | 1 |
| Ulcer disease | 531, 532, 533, 534 | K25, K26, K27, K28 | 1 |
| Mild liver disease | 07022,07023,07032,07033,07044,07054,0706,0709, 570, 571, 5733, 5734, 5738, 5739,V427, | B18, K73, K74, K700, K701, K702, K703, K709, K717, K713, K714, K715, K760, K762, K763, K764, K768, K769, Z944 | 1 |
| Diabetes without complications | 250, 2500, 2501, 2502, 2503, 2508, 2509, | E100, E101, E106, E108, E109, E110, E111, E116, E118, E119, E120, E121, E126, E128, E129, E130, E131, E136, E138, E139, E140, E141, E146, E148, E149 | 1 |
| Diabetes with complications | 2504, 2505, 2506, 2507 | E102, E103, E104, E105, E107, E112, E113, E114, E115, E117, E122, E123, E124, E125, E127, E132, E133, E134, E135, E137, E142, E143, E144, E145, E147 | 2 |
| Paraplegia and Hemiplegia | 3341, 342, 343, 3440, 3441, 3442, 3443, 3444, 3445, 3446, 3449 | G81, G82, G041, G114, G801, G802, G830, G831, G832, G833, G834, G839 | 2 |
| Renal disease | 40301, 40311, 40391, 40402, 40403, 40412, 40413, 40492, 40493, 582, 5830, 5831, 5832, 5834, 5836, 5837, 585, 586, 5880,V420,V451,V56, | N18, N19, N052, N053, N054, N055, N056, N057, N250, I120, I131, N032, N033, N034, N035, N036, N037, Z490, Z491, Z492, Z940, Z992 | 2 |
| Solid tumor | 140, 141, 142, 143, 144, 145, 146, 147, 148, 149, 150, 151, 152, 153, 154, 155, 156, 157, 158, 159, 160, 161, 162, 163, 164, 165, 170, 171, 172, 174, 175, 176, 179, 180, 181, 182, 183, 184, 185, 186, 187, 188, 189, 190, 191, 192, 193, 194 | C00, C01, C02, C03, C04, C05, C06, C07, C08, C09, C10, C11, C12, C13, C14, C15, C16, C17, C18, C19, C20, C21, C22, C23, C24, C25, C26, C30, C31, C32, C33, C34, C37, C38, C39, C40, C41, C43, C45, C46, C47, C48, C49, C50, C51, C52, C53, C54, C55, C56, C57, C58, C60, C61, C62, C63, C64, C65, C66, C67, C68, C69, C70, C71, C72, C73, C74, C75 | 2 |
| Leukemia | 204-207 | C91, C92, C93, C94, C95 | 2 |
| Lymphoma | 200-203, 275.59 | C81, C82, C83, C84, C85, C88, C90, C96 | 2 |
| Moderate or severe liver disease | 4560, 4561, 4562, 5722, 5723, 5724, 5728 | K704, K711, K721, K729, K765, K766, K767, I850, I859, I864, I982 | 3 |
| Metastatic carcinoma | 196, 197, 198, 195 | C76, C78, C79, C80 | 6 |

| **Disease** | **Operational definition** | **Note** |
| --- | --- | --- |
| severe kidney impairment | on dialysis or estimated GFR <30 mL/minute/1.73 m2 | Minimum of two outpatient visits or one hospitalization pertaining to the disease before the index date. (not including index date) |
| severe liver impairment | Cirrhosis: K740, K741, K742, K7460, K7469, K741, K742, K743, K744, K745, K7469, 5715, 5716  Hepatocellular carcinoma: C220, 1550  Liver transplantation: 75020B (Therapeutic Treatment code) | Minimum of two outpatient visits or one hospitalization pertaining to the disease before the index date. (not including index date) |

CCI, Charlson Comorbidity Index; COPD, chronic obstructive pulmonary disease; TMU, Taipei Medical University

**sTable 3. Basic Characteristics of COVID-19 Patients with Comorbidities Using Nirmatrelvir/Ritonavir or Not in 2022 before PSM**

|  | **Nirmatrelvir/Ritonavir^#^** | | | | **SMD** | **P value** |
| --- | --- | --- | --- | --- | --- | --- |
|  | **Yes** | | **No** | |  |  |
|  | **N=3478** | | **N=30940** | |  |  |
| **Gender** |  |  |  |  | 0.02 | 0.339 |
| Female | 1826 | 52.50% | 16508 | 53.35% |  |  |
| Male | 1652 | 47.50% | 14432 | 46.65% |  |  |
| **Age, mean±SD** | 67.52±14.01 | | 54.88±18.63 | | 0.71 | <.001 |
| 18~64 | 930 | 26.74% | 18577 | 60.04% |  |  |
| >=65 | 2548 | 73.26% | 12363 | 39.96% |  |  |
| **BMI, mean±SD** | 25.15±5.35 | | 26.35±6.30 | | 0.18 | <.001 |
| <30 | 2997 | 86.17% | 22733 | 73.47% |  |  |
| >=30 | 481 | 13.83% | 8207 | 26.53% |  |  |
| **Former patient at TMU’s hospitals** |  |  |  |  | 0.05 | 0.011 |
| No | 182 | 5.23% | 1961 | 6.34% |  |  |
| Yes | 3296 | 94.77% | 28979 | 93.66% |  |  |
| **Vaccinated at TMU’s hospitals** |  |  |  |  | 0.22 | <.001 |
| No | 2157 | 62.02% | 22392 | 72.37% |  |  |
| Yes | 1321 | 37.98% | 8548 | 27.63% |  |  |
| **Emergency Department** |  |  |  |  | 0.02 | 0.192 |
| No | 1158 | 33.29% | 10644 | 34.40% |  |  |
| Yes | 2320 | 66.71% | 20296 | 65.60% |  |  |
| **Telemedicine** |  |  |  |  | 0.22 | 0.011 |
| No | 3323 | 95.54% | 30652 | 99.07% |  |  |
| Yes | 155 | 4.46% | 288 | 0.93% |  |  |
| **CCI score, mean±SD** | 1.18±1.61 | | 0.81±1.42 | | 0.14 | <.001 |
| **Comorbidities** |  | |  | |  |  |
| Diabetes mellitus | 859 | 24.70% | 4512 | 14.58% | 0.26 | <.001 |
| Chronic kidney disease | 155 | 4.46% | 1727 | 5.58% | 0.05 | 0.006 |
| Cardiovascular disease (excluding hypertension) | 1124 | 32.32% | 9633 | 31.13% | 0.03 | 0.154 |
| Chronic pulmonary disease | 315 | 9.06% | 3104 | 10.03% | 0.03 | 0.068 |
| Immunodeficiency or immunosuppression | 119 | 3.42% | 1264 | 4.09% | 0.04 | 0.059 |
| Malignancy | 272 | 7.82% | 1619 | 5.23% | 0.11 | <.001 |
| Tuberculosis | 7 | 0.20% | 97 | 0.31% | 0.02 | 0.253 |
| Chronic liver diseases | 91 | 2.62% | 888 | 2.87% | 0.02 | 0.394 |
| Mental disease | 174 | 5.00% | 1640 | 5.30% | 0.01 | 0.456 |
| Dementia | 176 | 5.06% | 1055 | 3.41% | 0.08 | <.001 |

BMI, body mass index; CCI, Charlson Comorbidity Index; N, number; PSM, propensity score matching; SD, standard deviation; SMD, standardized mean differences; TMU, Taipei Medical University

**sTable 4. Basic Characteristics of COVID-19 Patients with Comorbidities Using Molnupiravir or Not in 2022 before PSM**

|  | **Molnupiravir** | | | | **SMD** | **P value** |
| --- | --- | --- | --- | --- | --- | --- |
|  | **Yes** | | **No** | |  |  |
|  | **N=1199** | | **N=30940** | |  |  |
| **Gender** |  |  |  |  | 0.11 | <.001 |
| Female | 572 | 47.71% | 16508 | 53.35% |  |  |
| Male | 627 | 52.29% | 14432 | 46.65% |  |  |
| **Age, mean±SD** | 72.92±14.16 | | 55.95±18.82 | | 1.02 | <.001 |
| 18~64 | 275 | 22.94% | 18577 | 60.04% |  |  |
| >=65 | 924 | 77.06% | 12363 | 39.96% |  |  |
| **BMI, mean±SD** | 24.73±5.08 | | 26.22±6.25 | | 0.26 | <.001 |
| <30 | 1053 | 87.82% | 22733 | 73.47% |  |  |
| >=30 | 146 | 12.18% | 8207 | 26.53% |  |  |
| **Former patient at TMU’s hospitals** |  |  |  |  | 0.05 | 0.080 |
| No | 61 | 5.09% | 1961 | 6.34% |  |  |
| Yes | 1138 | 94.91% | 28979 | 93.66% |  |  |
| **Vaccinated at TMU’s hospitals** |  |  |  |  | 0.21 | <.001 |
| No | 748 | 62.39% | 22392 | 72.37% |  |  |
| Yes | 451 | 37.61% | 8548 | 27.63% |  |  |
| **Emergency Department** |  |  |  |  | 0.03 | 0.270 |
| No | 394 | 32.86% | 10644 | 34.40% |  |  |
| Yes | 805 | 67.14% | 20296 | 65.60% |  |  |
| **Telemedicine** |  |  |  |  | 0.30 | <.001 |
| No | 1119 | 93.33% | 30652 | 99.07% |  |  |
| Yes | 80 | 6.67% | 288 | 0.93% |  |  |
| **CCI score, mean±SD** | 2.75±2.60 | | 1.03±1.76 | | 0.77 | <.001 |
| **Comorbidities** |  | |  | |  |  |
| Diabetes mellitus | 430 | 35.86% | 4512 | 14.58% | 0.51 | <.001 |
| Chronic kidney disease | 395 | 32.94% | 1727 | 5.58% | 0.74 | <.001 |
| Cardiovascular disease (excluding hypertension) | 680 | 56.71% | 9633 | 31.13% | 0.53 | <.001 |
| Chronic pulmonary disease | 152 | 12.68% | 3104 | 10.03% | 0.08 | 0.003 |
| Immunodeficiency or immunosuppression | 54 | 4.50% | 1264 | 4.09% | 0.02 | 0.474 |
| Malignancy | 118 | 9.84% | 1619 | 5.23% | 0.18 | <.001 |
| Tuberculosis | 8 | 0.67% | 97 | 0.31% | 0.05 | 0.035 |
| Chronic liver diseases | 50 | 4.17% | 888 | 2.87% | 0.07 | 0.009 |
| Mental disease | 87 | 7.26% | 1640 | 5.30% | 0.08 | 0.003 |
| Dementia | 164 | 13.68% | 1055 | 3.41% | 0.37 | <.001 |

BMI, body mass index; CCI, Charlson Comorbidity Index; N, number; PSM, propensity score matching; SD, standard deviation; SMD, standardized mean differences; TMU, Taipei Medical University

**sTable 5. Basic characteristics of COVID-19 Patients with Comorbidities Using Nirmatrelvir/Ritonavir or Molnupiravir in 2022 before PSM**

|  | **COVID drugs** | | | | **SMD** | **P value** |
| --- | --- | --- | --- | --- | --- | --- |
|  | **Nirmatrelvir/Ritonavir** | | **Molnupiravir** | |  |  |
|  | **N=3478** | | **N=1199** | |  |  |
| **Gender** |  |  |  |  | 0.10 | 0.004 |
| Female | 1826 | 52.50% | 572 | 47.71% |  |  |
| Male | 1652 | 47.50% | 627 | 52.29% |  |  |
| **Age, mean±SD** | 67.70±13.99 | | 72.92±14.16 | | 0.37 | <.001 |
| 18~64 | 930 | 26.74% | 275 | 22.94% |  |  |
| >=65 | 2548 | 73.26% | 924 | 77.06% |  |  |
| **BMI, mean±SD** | 25.15±5.35 | | 24.73±5.08 | | 0.08 | 0.002 |
| <30 | 2997 | 86.17% | 1053 | 87.82% |  |  |
| >=30 | 481 | 13.83% | 146 | 12.18% |  |  |
| **Former patient at TMU’s hospitals** |  |  |  |  | 0.01 | 0.845 |
| No | 182 | 5.23% | 61 | 5.09% |  |  |
| Yes | 3296 | 94.77% | 1138 | 94.91% |  |  |
| **Vaccinated at TMU’s hospitals** |  |  |  |  | 0.01 | 0.821 |
| No | 2157 | 62.02% | 748 | 62.39% |  |  |
| Yes | 1321 | 37.98% | 451 | 37.61% |  |  |
| **Emergency Department** |  |  |  |  | 0.01 | 0.783 |
| No | 1158 | 33.29% | 394 | 32.86% |  |  |
| Yes | 2320 | 66.71% | 805 | 67.14% |  |  |
| **Telemedicine** |  |  |  |  | 0.10 | 0.003 |
| No | 3323 | 95.54% | 1119 | 93.33% |  |  |
| Yes | 155 | 4.46% | 80 | 6.67% |  |  |
| **CCI score, mean±SD** | 1.18±1.61 | | 2.75±2.60 | | 0.67 | <.001 |
| **Comorbidities** |  | |  | |  |  |
| Diabetes mellitus | 859 | 24.70% | 430 | 35.86% | 0.24 | <.001 |
| Chronic kidney disease | 155 | 4.46% | 395 | 32.94% | 0.78 | <.001 |
| Cardiovascular disease (excluding hypertension) | 1124 | 32.32% | 680 | 56.71% | 0.51 | <.001 |
| Chronic pulmonary disease | 315 | 9.06% | 152 | 12.68% | 0.12 | <.001 |
| Immunodeficiency or immunosuppression | 119 | 3.42% | 54 | 4.50% | 0.06 | 0.087 |
| Malignancy | 272 | 7.82% | 118 | 9.84% | 0.07 | 0.029 |
| Tuberculosis | 7 | 0.20% | 8 | 0.67% | 0.07 | 0.014 |
| Chronic liver diseases | 91 | 2.62% | 50 | 4.17% | 0.09 | 0.007 |
| Mental disease | 174 | 5.00% | 87 | 7.26% | 0.09 | 0.003 |
| Dementia | 176 | 5.06% | 164 | 13.68% | 0.30 | <.001 |

BMI, body mass index; CCI, Charlson Comorbidity Index; N, number; PSM, propensity score matching; SD, standard deviation; SMD, standardized mean differences; TMU, Taipei Medical University

**sTable 6. Basic Characteristics of COVID-19 patients with Comorbidities Using Nirmatrelvir/Ritonavir or Not in 2022 after PSM**

|  | **Nirmatrelvir/Ritonavir**^#^ | | | | **SMD** | **P value** |
| --- | --- | --- | --- | --- | --- | --- |
|  | **Yes** | | **No** | |  |  |
|  | **N=3469** | | **N=13465** | |  |  |
| **Gender** |  |  |  |  | 0.01 | 0.750 |
| Female | 1821 | 52.49% | 7109 | 52.80% |  |  |
| Male | 1648 | 47.51% | 6356 | 47.20% |  |  |
| **Age, mean±SD** | 67.66±13.98 | | 67.90±14.55 | | 0.02 | 0.355 |
| . 18~64 | 930 | 26.81% | 4265 | 31.67% |  |  |
| >=65 | 2539 | 73.19% | 9200 | 68.33% |  |  |
| **BMI, mean±SD** | 25.15±5.36 | | 25.11±5.33 | | 0.01 | 0.704 |
| <30 | 2989 | 86.16% | 11369 | 84.43% |  |  |
| >=30 | 480 | 13.84% | 2096 | 15.57% |  |  |
| **Former patient at TMU’s hospitals** |  |  |  |  | 0.02 | 0.328 |
| No | 182 | 5.25% | 764 | 5.67% |  |  |
| Yes | 3287 | 94.75% | 12701 | 94.33% |  |  |
| **Vaccinated at TMU’s hospitals** |  |  |  |  | 0.04 | 0.026 |
| No | 2154 | 62.09% | 8635 | 64.13% |  |  |
| Yes | 1315 | 37.91% | 4830 | 35.87% |  |  |
| **Emergency Department** |  |  |  |  | 0.02 | 0.396 |
| No | 1149 | 33.12% | 4358 | 32.37% |  |  |
| Yes | 2320 | 66.88% | 9107 | 67.63% |  |  |
| **Telemedicine** |  |  |  |  | 0.13 | <.001 |
| No | 3323 | 95.79% | 13197 | 98.01% |  |  |
| Yes | 146 | 4.21% | 268 | 1.99% |  |  |
| **CCI score, mean±SD** | 1.28±1.73 | | 1.27±1.92 | | 0.01 | 0.883 |
| **Comorbidities** |  | |  | |  |  |
| Diabetes mellitus | 855 | 24.65% | 3166 | 23.51% | 0.03 | 0.162 |
| Chronic kidney disease | 155 | 4.47% | 674 | 5.01% | 0.03 | 0.191 |
| Cardiovascular disease (excluding hypertension) | 1118 | 32.23% | 4322 | 32.10% | 0.00 | 0.884 |
| Chronic pulmonary disease | 315 | 9.08% | 1201 | 8.92% | 0.01 | 0.767 |
| Immunodeficiency or immunosuppression | 119 | 3.43% | 450 | 3.34% | 0.00 | 0.797 |
| Malignancy | 272 | 7.84% | 1000 | 7.43% | 0.02 | 0.409 |
| Tuberculosis | 7 | 0.20% | 28 | 0.21% | 0.00 | 0.943 |
| Chronic liver diseases | 91 | 2.62% | 349 | 2.59% | 0.00 | 0.918 |
| Mental disease | 174 | 5.02% | 655 | 4.86% | 0.01 | 0.713 |
| Dementia | 174 | 5.02% | 675 | 5.01% | 0.00 | 0.995 |

# Using 1: up to 4 propensity score matching with matching variables including all variables in table S6.

BMI, body mass index; CCI, Charlson Comorbidity Index; N, number; PSM, propensity score matching; SD, standard deviation; SMD, standardized mean differences; TMU, Taipei Medical University

**sTable 7. Basic Characteristics of COVID-19 Patients with Comorbidities Using Molnupiravir or Not in 2022 after PSM**

|  | **Molnupiravir**^#^ | | | | **SMD** | **P value** |
| --- | --- | --- | --- | --- | --- | --- |
|  | **Yes** | | **No** | |  |  |
|  | **N=1191** | | **N=4606** | |  |  |
| **Gender** |  |  |  |  | 0.02 | 0.515 |
| Female | 568 | 47.69% | 2148 | 46.63% |  |  |
| Male | 623 | 52.31% | 2458 | 53.37% |  |  |
| **Age, mean±SD** | 72.87±14.19 | | 73.79±13.42 | | 0.07 | 0.043 |
| 18~64 | 275 | 23.09% | 952 | 20.67% |  |  |
| >=65 | 916 | 76.91% | 3654 | 79.33% |  |  |
| **BMI, mean±SD** | 24.72±5.09 | | 24.58±4.87 | | 0.03 | 0.362 |
| <30 | 1047 | 87.91% | 4091 | 88.82% |  |  |
| >=30 | 144 | 12.09% | 515 | 11.18% |  |  |
| **Former patient at TMU’s hospitals** |  |  |  |  | 0.02 | 0.464 |
| No | 61 | 5.12% | 261 | 5.67% |  |  |
| Yes | 1130 | 94.88% | 4345 | 94.33% |  |  |
| **Vaccinated at TMU’s hospitals** |  |  |  |  | 0.01 | 0.779 |
| No | 743 | 62.38% | 2853 | 61.94% |  |  |
| Yes | 448 | 37.62% | 1753 | 38.06% |  |  |
| **Emergency Department** |  |  |  |  | 0.03 | 0.031 |
| No | 386 | 32.41% | 1345 | 29.20% |  |  |
| Yes | 805 | 67.59% | 3261 | 70.80% |  |  |
| **Telemedicine** |  |  |  |  | 0.07 | <.001 |
| No | 1119 | 93.95% | 4432 | 96.22% |  |  |
| Yes | 72 | 6.05% | 174 | 3.78% |  |  |
| **CCI score, mean±SD** | 2.74±2.59 | | 2.58±2.69 | | 0.06 | 0.074 |
| **Comorbidities** |  | |  | |  |  |
| Diabetes mellitus | 424 | 35.60% | 1664 | 36.13% | 0.01 | 0.736 |
| Chronic kidney disease | 387 | 32.49% | 1251 | 27.16% | 0.12 | <.001 |
| Cardiovascular disease (excluding hypertension) | 673 | 56.51% | 2651 | 57.56% | 0.02 | 0.514 |
| Chronic pulmonary disease | 151 | 12.68% | 579 | 12.57% | 0.00 | 0.920 |
| Immunodeficiency or immunosuppression | 54 | 4.53% | 204 | 4.43% | 0.00 | 0.876 |
| Malignancy | 118 | 9.91% | 442 | 9.60% | 0.01 | 0.746 |
| Tuberculosis | 8 | 0.67% | 27 | 0.59% | 0.01 | 0.734 |
| Chronic liver diseases | 50 | 4.20% | 178 | 3.86% | 0.02 | 0.598 |
| Mental disease | 86 | 7.22% | 305 | 6.62% | 0.02 | 0.463 |
| Dementia | 162 | 13.60% | 590 | 12.81% | 0.02 | 0.468 |

# Using 1: up to 4 propensity score matching with matching variables including all variables in table S7

BMI, body mass index; CCI, Charlson Comorbidity Index; N, number; PSM, propensity score matching; SD, standard deviation; SMD, standardized mean differences; TMU, Taipei Medical University

**sTable 8. Variance Ratios of Baseline Characteristics of COVID-19 Patients with Comorbidities Using Nirmatrelvir/Ritonavir, Molnupiravir or Not**

|  | Variance Ratios | | |
| --- | --- | --- | --- |
| Variables | Nirmatrelvir/Ritonavir vs  none | Molnupiravir  vs  none | Nirmatrelvir/Ritonavir  vs  Molnupiravir |
| Age | 0.9236 | 1.1193 | 0.7916 |
| BMI | 1.0114 | 1.0903 | 0.8038 |
| CCI score | 0.8127 | 0.9314 | 0.7537 |
| Gender | 1.0006 | 1.0024 | 1.0001 |
| Diabetes mellitus | 1.0327 | 0.9935 | 0.9304 |
| Chronic kidney disease | 0.8977 | 1.1088 | 0.6061 |
| Cardiovascular disease (excluding hypertension) | 1.0021 | 1.006 | 1.0145 |
| Chronic pulmonary disease | 1.0163 | 1.0073 | 0.8802 |
| Immunodeficiency or immunosuppression | 1.0255 | 1.0226 | 0.7202 |
| Malignancy | 1.051 | 1.0289 | 0.9968 |
| Tuberculosis | 0.9704 | 1.1449 | 0.4658 |
| Chronic liver diseases | 1.0118 | 1.0826 | 0.8233 |
| Mental disease | 1.0295 | 1.0835 | 1.1513 |
| Dementia | 1.0005 | 1.0522 | 0.9826 |
| Telemedicine | 2.0667 | 1.5626 | 0.9586 |
| Emergency Department | 1.0119 | 1.0596 | 0.9557 |
| Vaccinated at TMU’s hospitals | 1.0232 | 0.9954 | 0.9242 |
| Former patient at TMU’s hospitals | 0.9288 | 0.9091 | 1.3699 |

BMI, body mass index; CCI, Charlson Comorbidity Index; TMU, Taipei Medical University

**sFigure 1. Density Plots of Propensity Scores Before and After Matching in COVID-19 Patients Using Nirmatrelvir/Ritonavir or Molnupiravir in 2022**

| **(A)**  **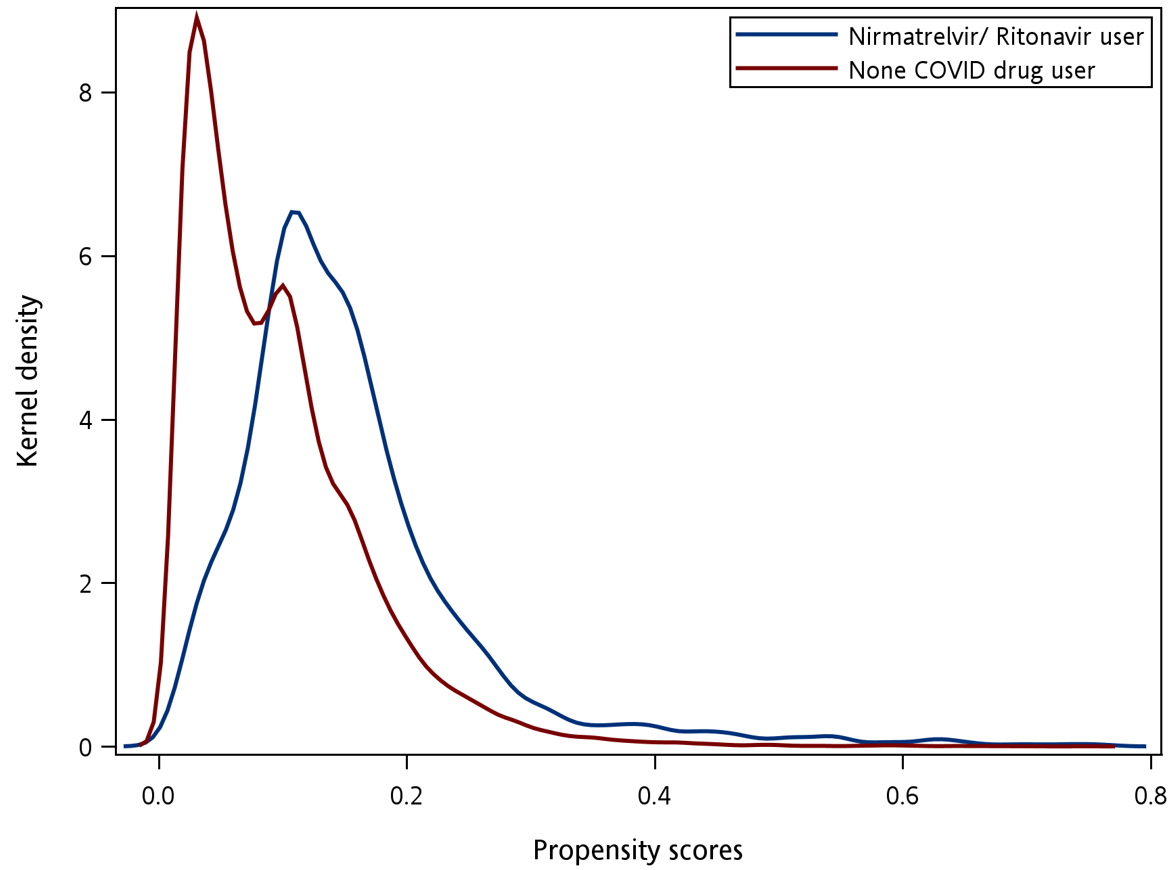** | **(B)**  **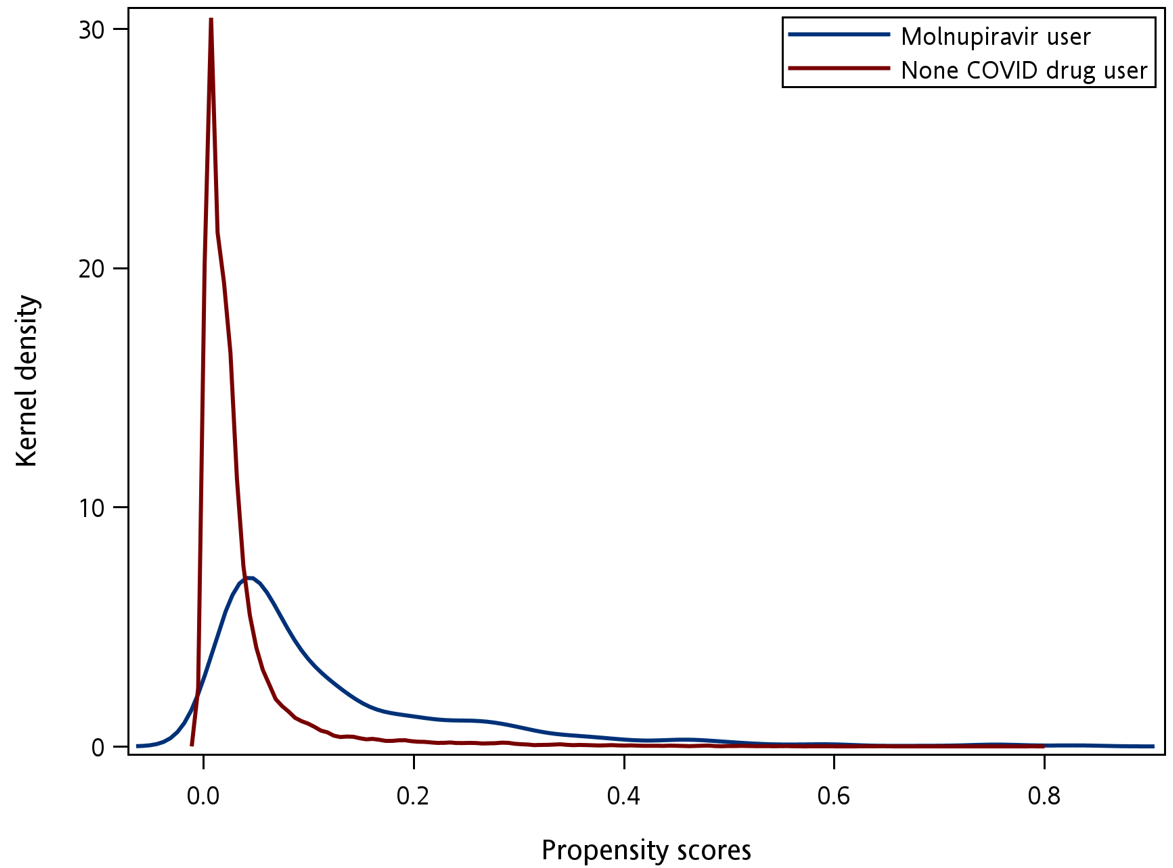** | **(C)**  **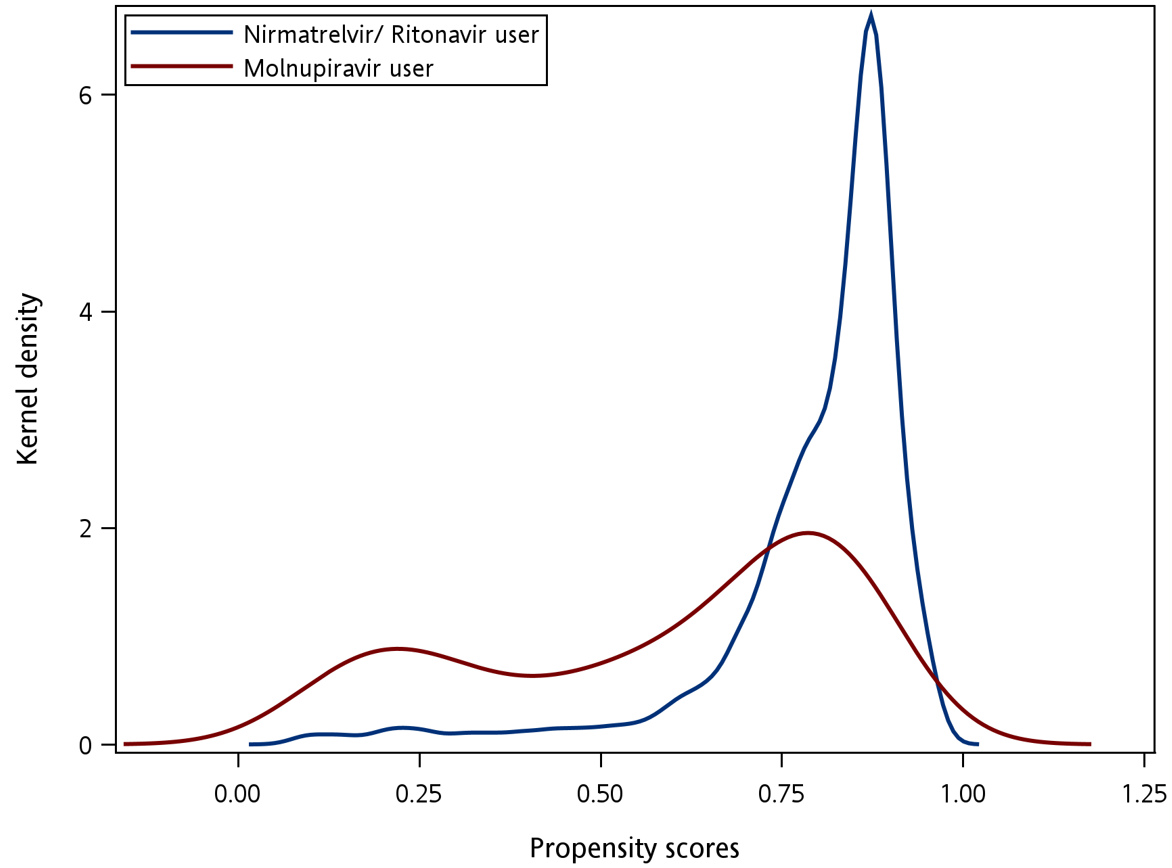** |
| --- | --- | --- |
| **(D)**  **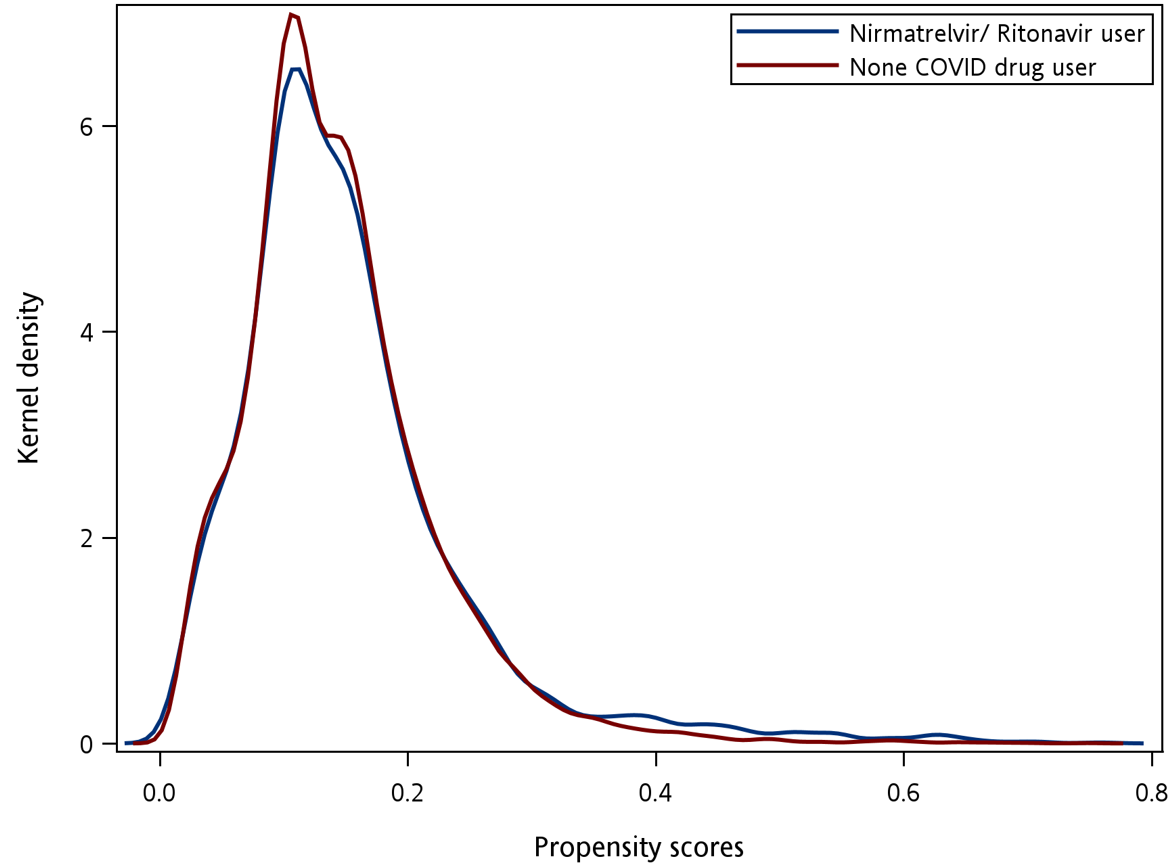** | **(E)**  **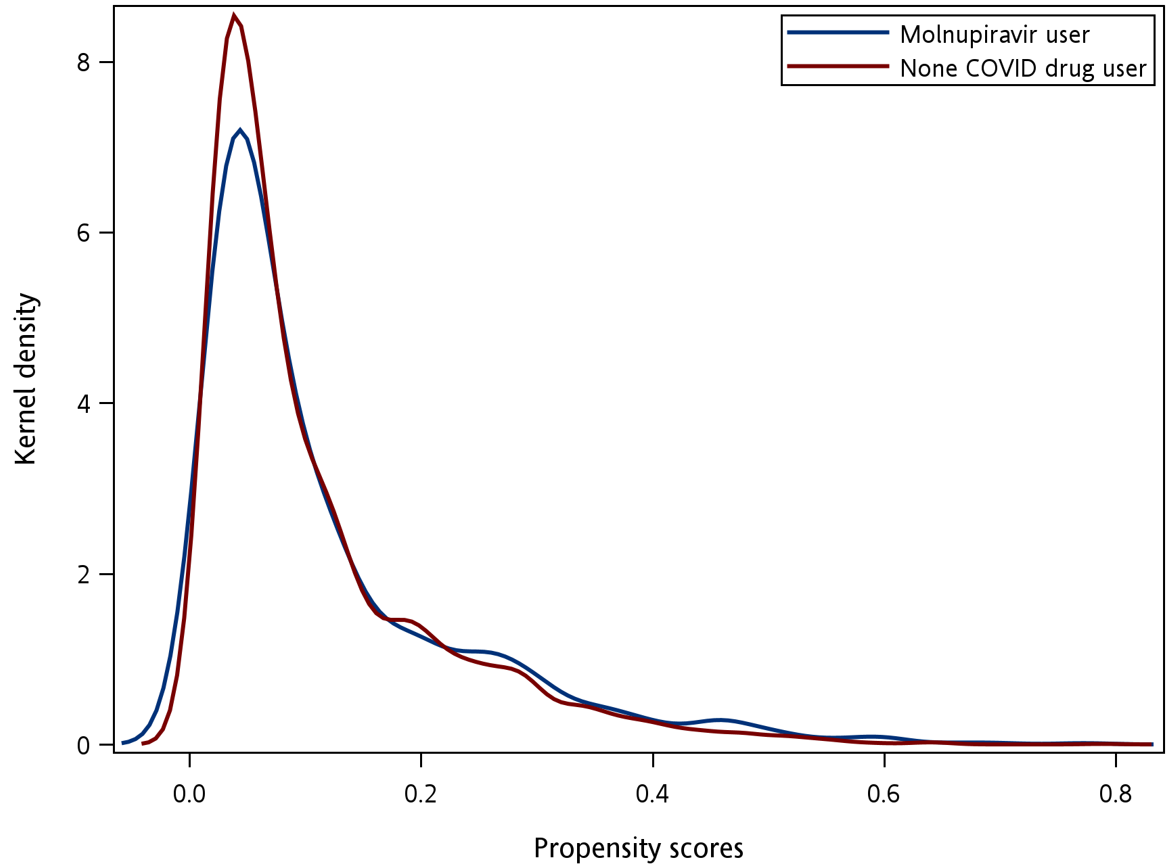** | **(F)**  **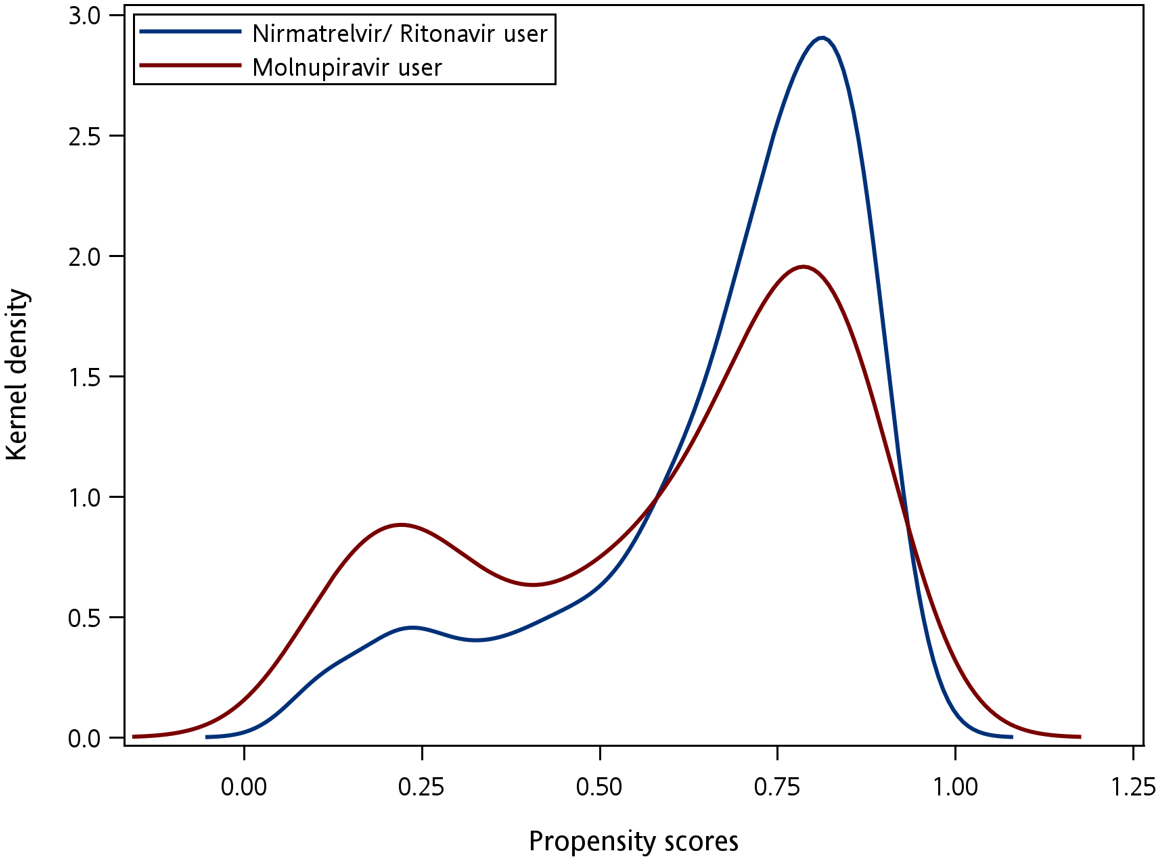** |

(A) Basic Characteristics of COVID-19 Patients with Comorbidities Using Nirmatrelvir/Ritonavir or Not in 2022 before PSM, (B) Basic Characteristics of COVID-19 Patients with Comorbidities Using Molnupiravir or Not in 2022 before PSM, (C) Basic Characteristics of COVID-19 Patients with Comorbidities Using Nirmatrelvir/Ritonavir or Molnupiravir in 2022 before PSM, (D) Basic Characteristics of COVID-19 Patients with Comorbidities Using Nirmatrelvir/Ritonavir or Not in 2022 after PSM, (E) Basic Characteristics of COVID-19 Patients with Comorbidities Using Molnupiravir or Not in 2022 after PSM and (F) Basic Characteristics of COVID-19 Patients with Comorbidities Using Nirmatrelvir/Ritonavir or Molnupiravir in 2022 after PSM. The plot A to C illustrated significant differences in score distributions between the two groups before matching, whereas the plot D to F demonstrated that the distributions nearly completely overlap after matching.

PSM, propensity score matching

**sFigure 2. LOVE Plots Demonstrated the Absolute Standardized Mean Differences on the Matching Variables Between Nirmatrelvir/Ritonavir and Molnupiravir**

| **(A)**  **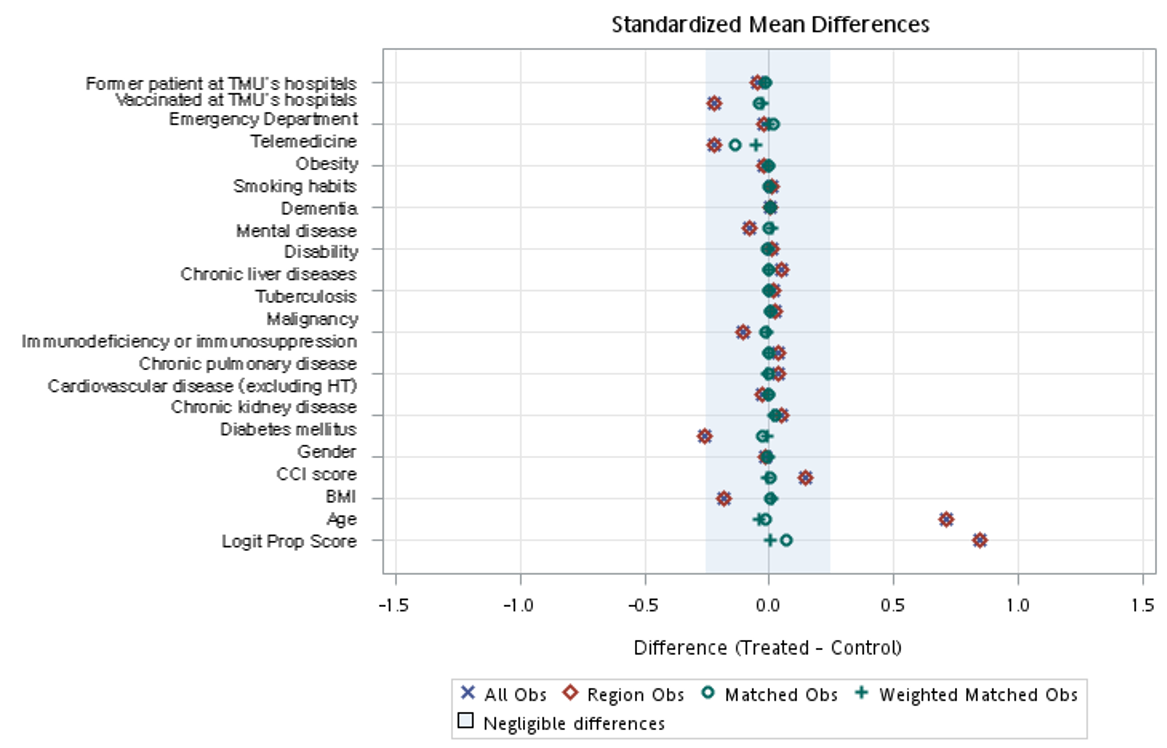** | **(B)**  **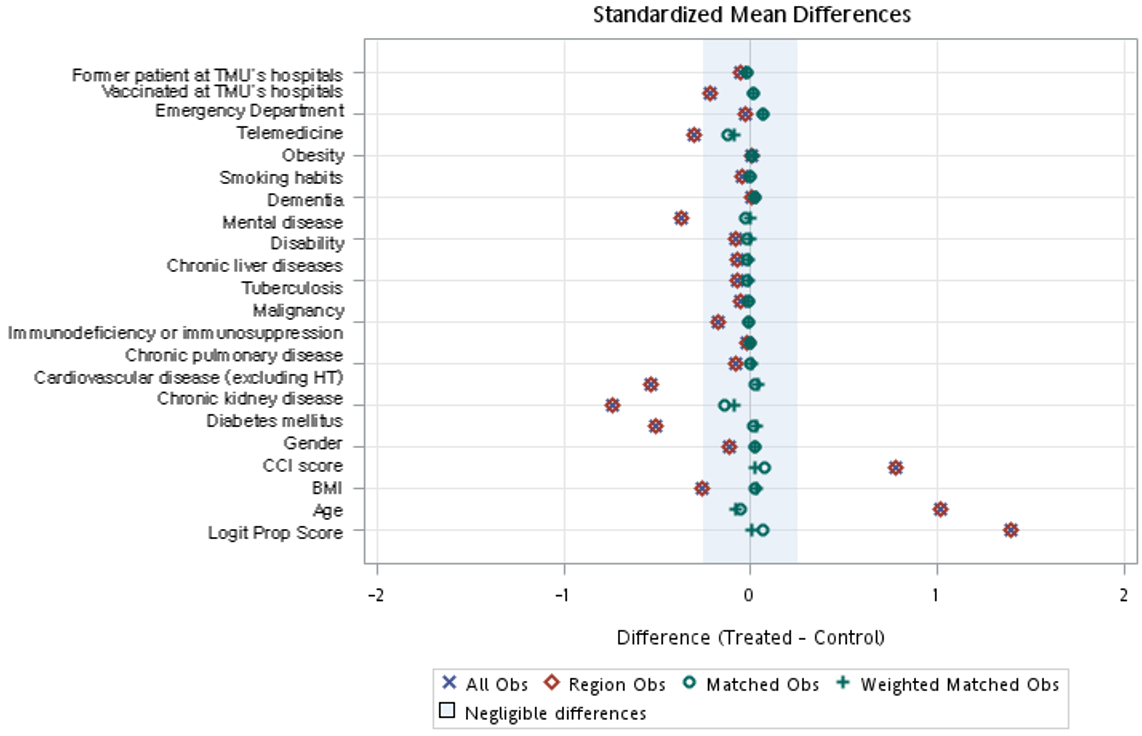** | **(C)**  **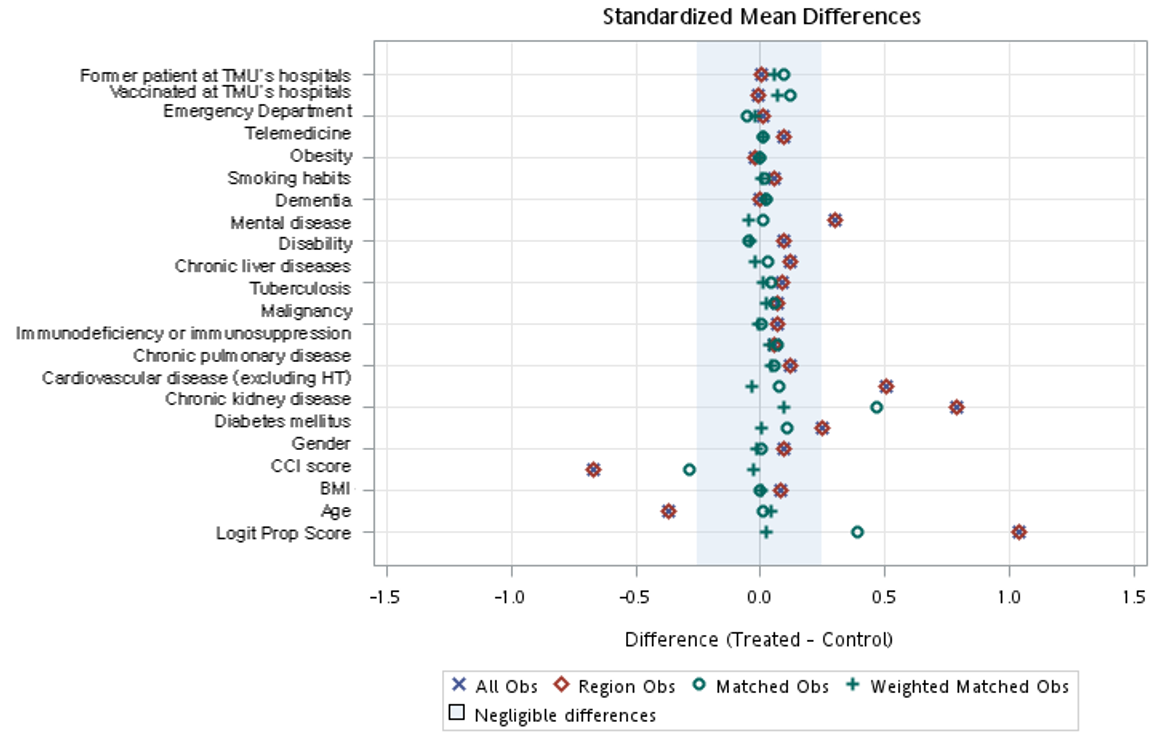** |
| --- | --- | --- |

(A) COVID-19 Patient Using Nirmatrelvir/Ritonavir or Not, (B) COVID-19 Patient Using Molnupiravir or Not, and (C) COVID-19 Patient Using Nirmatrelvir/Ritonavir or Molnupiravir.

BMI, body mass index; CCI, Charlson Comorbidity Index; Obs, Observation; TMU, Taipei Medical University

**sFigure 3. Risk of Invasive Ventilation in COVID-19 Patients with Comorbidities Using Nirmatrelvir/Ritonavir or not in 2022**

**[patients with inpatient prescription were excluded; outcomes within 30 days after COVID diagnosis]**


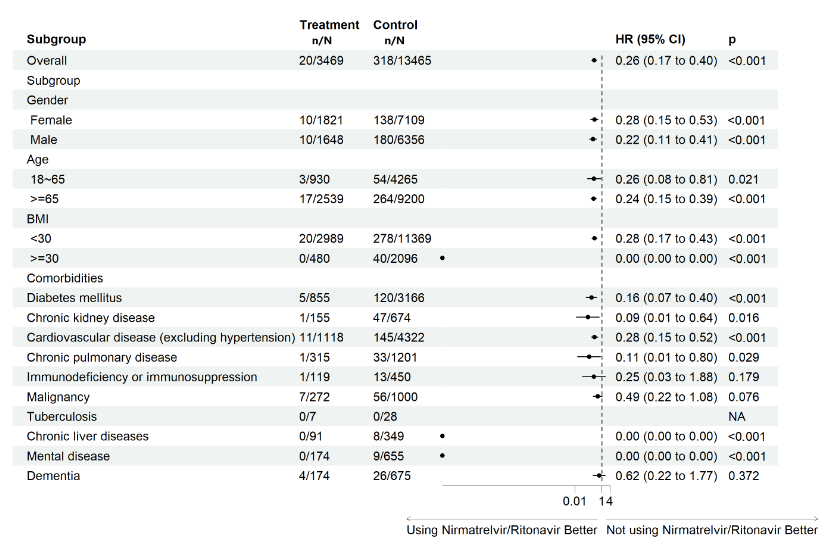


Adjusted by age, BMI, telemedicine, and vaccinated or not at TMU’s hospitals

Treatment group represents group of nirmatrelvir/ritonavir; control group represents group of patients who did not receive any antivirus treatment

BMI, body mass index; CI, confidence interval; HR, hazard ratio; n, number (event number); N, number (treatment number); NA, not applicable; TMU: Taipei Medical University

**sFigure 4. Risk of Invasive Ventilation in COVID-19 Patients with Comorbidities Using Molnupiravir or not in 2022**

**[patients with inpatient prescription were excluded; outcomes within 30 days after COVID diagnosis]**


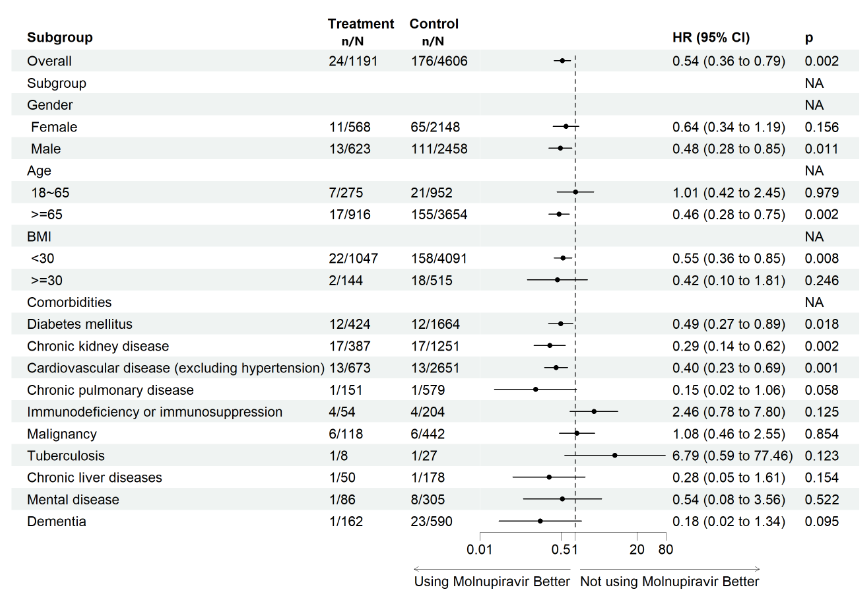


Adjusted by age, BMI, chronic kidney disease, telemedicine, and COVID diagnosed in the emergency department or not

Treatment group represents group of molnupiravir; control group represents group of patients who did not receive any antivirus treatment

BMI, body mass index; CI, confidence interval; HR, hazard ratio; n, number (event number); N, number (treatment number); NA, not applicable

**sFigure 5. Risk of Mortality in COVID-19 Patients with Comorbidities Using Nirmatrelvir/Ritonavir or not in 2022**

**[patients with inpatient prescription were excluded; outcomes within 30 days after COVID diagnosis]**


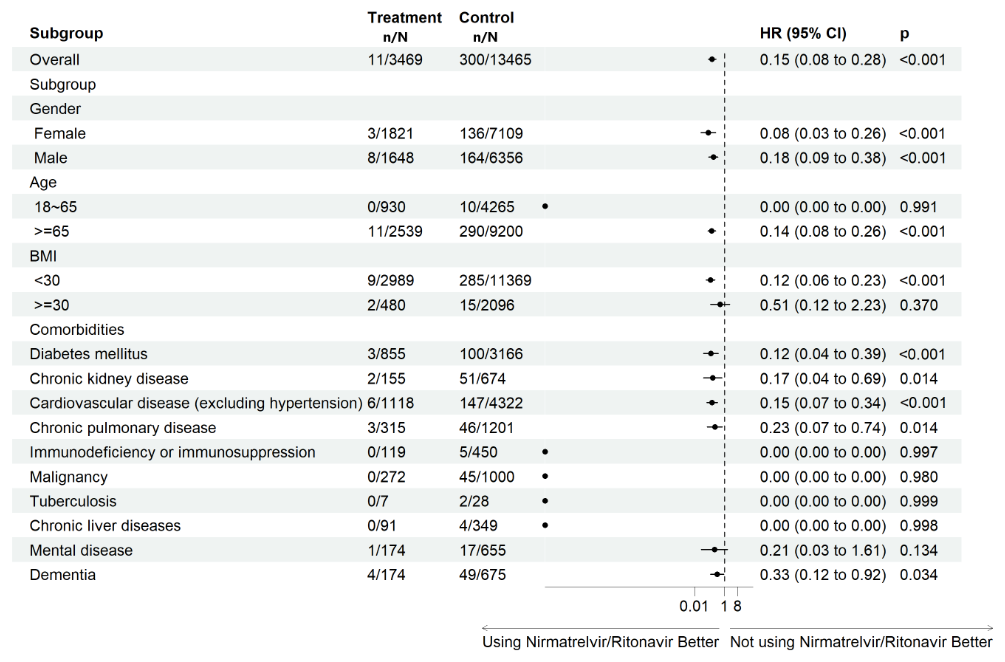


Adjusted by age, BMI, telemedicine, and vaccinated or not at TMU’s hospitals

Treatment group represents group of nirmatrelvir/ritonavir; control group represents group of patients who did not receive any antivirus treatment

BMI, body mass index; CI, confidence interval; HR, hazard ratio; n, number (event number); N, number (treatment number); TMU: Taipei Medical University

**sFigure 6. Risk of Mortality in COVID-19 Patients with Comorbidities Using Molnupiravir or not in 2022**

**[patients with inpatient prescription were excluded; outcomes within 30 days after COVID diagnosis]**


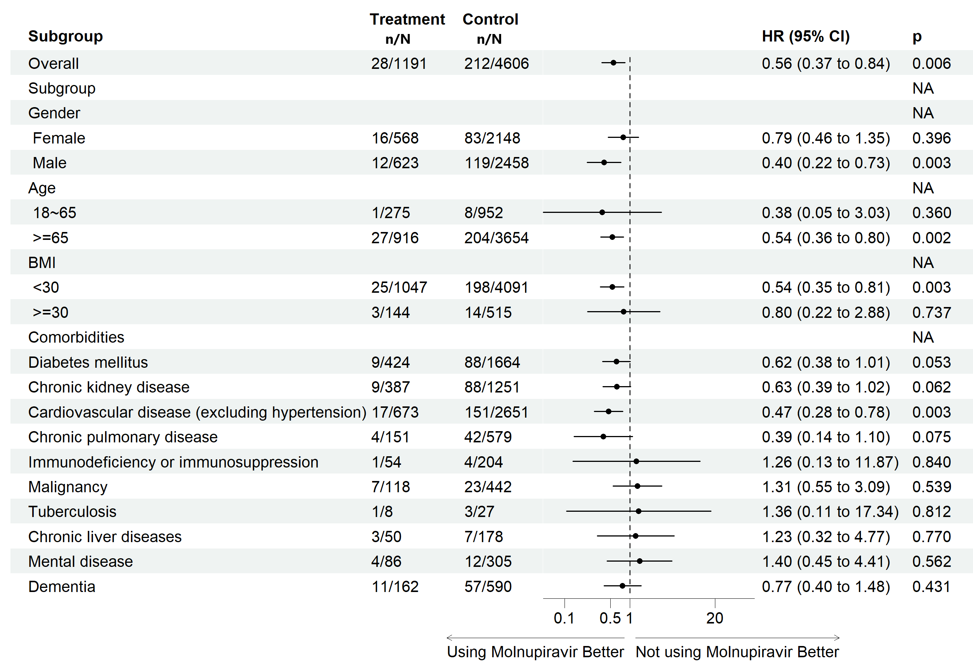


Adjusted by age, BMI, chronic kidney disease, telemedicine, and COVID diagnosed in the emergency department or not

Treatment group represents group of molnupiravir; control group represents group of patients who did not receive any antivirus treatment

BMI, body mass index; CI, confidence interval; HR, hazard ratio; n, number (event number); N, number (treatment number); NA, not applicable
